# COF-300 synthesis and colloidal stabilization with substituted benzoic acids†

**DOI:** 10.1039/d3ra02202a

**Published:** 2023-05-12

**Authors:** Woojung Ji, Dean M. Kim, Brendan M. Posson, Kyla J. Carlson, Alison C. Chew, Alyssa J. Chew, Meherin Hossain, Alexis F. Mojica, Sachi M. Ottoes, Donna V. Tran, Matthew W. Greenberg, Leslie S. Hamachi

**Affiliations:** a Department of Chemistry, University of Michigan Ann Arbor 48109 MI USA; b Department of Chemistry and Biochemistry, California Polytechnic State University San Luis Obispo 93407 CA USA hamachi@calpoly.edu; c Department of Materials Engineering, California Polytechnic State University San Luis Obispo CA 93407 USA; d Department of Chemistry and Biochemistry, Bard College Annandale-on-Hudson NY 12504 USA

## Abstract

Colloidal covalent organic framework (COF) synthesis enables morphological control of crystallite size and shape. Despite numerous examples of 2D COF colloids with various linkage chemistries, 3D imine-linked COF colloids are more challenging synthetic targets. Here we report a rapid (15 min–5 day) synthesis of hydrated COF-300 colloids ranging in length (251 nm–4.6 μm) with high crystallinity and moderate surface areas (150 m^2^ g^−1^). These materials are characterized by pair distribution function analysis, which is consistent with the known average structure for this material alongside different degrees of atomic disorder at different length scales. Additionally, we investigate a series of *para*-substituted benzoic acid catalysts, finding that 4-cyano and 4-fluoro substituted benzoic acids produce the largest COF-300 crystallites with lengths of 1–2 μm. *In situ* dynamic light scattering experiments are used to assess time to nucleation in conjunction with ^1^H NMR model compound studies to probe the impact of catalyst acidity on the imine condensation equilibrium. We observe cationically stabilized colloids with a zeta potential of up to +14.35 mV in benzonitrile as a result of the carboxylic acid catalyst protonating surface amine groups. We leverage these surface chemistry insights to synthesize small COF-300 colloids using sterically hindered diortho-substituted carboxylic acid catalysts. This fundamental study of COF-300 colloid synthesis and surface chemistry will provide new insights into the role of acid catalysts both as imine condensation catalysts and as colloid stabilizing agents.

## Abstract 2

Colloidal COF-300 synthesis with substituted benzoic acid catalysts demonstrates that acidity plays a role in reaction kinetics and the resulting particle's colloidal stability.

## Graphical abstract

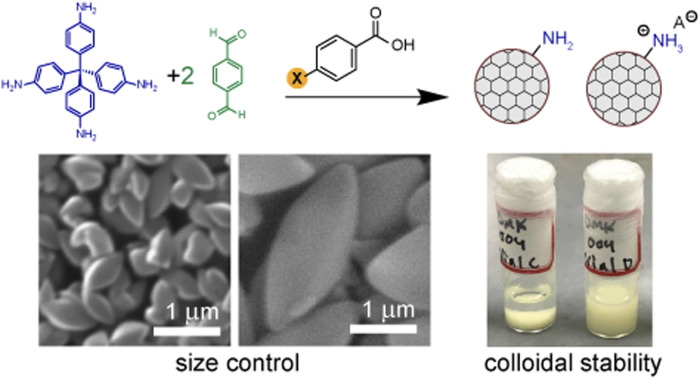

## Introduction

Covalent organic frameworks (COFs) are crystalline polymers featuring high porosity^2,3^ that enables applications including gas storage,^4^ catalysis,^5,6^ and pollutant remediation for both metal-ion^7,8^ and organic pollutants.^9–12^ These polymers are highly modular with pore sizes and shapes dictated by monomer choice.^13^ Based on monomer geometry, COFs are further divided into two dimensional (2D) and three dimensional (3D) network topologies. A majority of synthetic efforts focus on 2D COFs comprised of covalently linked planar sheets stacked in three dimensions *via* π–π interactions. 3D COFs that possess covalent bonds extending in all three dimensions are more challenging synthetic targets due to the absence of π–π interactions to drive the crystallization process.^14^

Most successful COF syntheses have optimized reaction conditions for covalent bond reversibility which enables “error correction” in a growing COF.^15^ These simultaneous bond formation and reversion processes are promoted *via* the addition of a mono-functional modulator compound that slows down crystallization kinetics, resulting in the production of thermodynamically favored crystalline products.^1,16^ To date, four notable syntheses of single crystalline 3D COFs employing modulator strategies have been reported as bulk powders^1,17,18^ or as colloids.^19^ Modulators have also been employed for shape and morphology control in 2D COF systems.^20–22^ Although modulator strategies have demonstrated success in increasing crystalline domain sizes, particle size, and shape, COF growth typically occurs under heterogeneous, solvothermal reaction conditions. Using these solvothermal methods, precipitation irreversibly traps kinetic defects, preventing formation of the thermodynamically favored crystalline material. Homogeneous initial reaction conditions including the use of protected monomers^23^ or colloidal synthesis strategies^19,24,25^ seek to solve this problem and have been employed towards the growth of high-quality COFs. However, despite several reports on the synthesis of colloidal 3D boroxine-linked COF colloids^19^ and 2D boronate ester-,^21,24,25^ 2D boroxine-,^19^ and 2D imine-linked COF colloids,^22,26,27^ 3D imine-linked COF colloids have only recently been synthesized.^28^ High-quality 3D imine-linked COFs are desirable synthetic targets due to their small pore sizes and greater hydrolytic stability compared to their boron-containing counterparts.^14,29^ Colloidal control over 3D COF crystallite size and shape will enable further optimization of material performance.^30^

Related porous framework literature demonstrates the impact of control over crystallite size and shape. For example, nanometer-sized metal organic frameworks (nanoMOFs) demonstrate higher catalytic activity,^31^ faster adsorption kinetics,^32^ and enhanced dye adsorption^33^ compared to their bulk counterparts. This enhanced performance is attributed to mass transport limitations in the bulk. Studies on colloidal COF synthesis to access smaller particle sizes could similarly produce COF materials with enhanced capabilities. In some instances, as for colloidal COF-300,^28^ the reaction concentration is varied in order to tune particle size. However, changes in concentration can also affect the crystallinity of the material. Another way to control size and shape of porous framework materials relies on surface chemistry and functionalization. Although many efforts have focused on seeded growth methods^25,26^ or post-synthetic functionalization or processing methods,^28,34–36^ a more thorough understanding of colloidal COF surface chemistry could provide another means to control COF particle size. Indeed, recent reports have explored the surface chemistry of unreacted pendant amines at the surfaces of COF-300 colloids.^28^ Another notable example achieves size control of a 2D COF using different amounts of acetic acid catalyst.^37^ However in this example, it is unclear if the effect on size is from enhanced dynamic bond exchange resulting in higher error correction or if it is from an interaction of the acid catalyst with surface amines. A more thorough understanding of colloidal 3D COF surface chemistry will both enable control of particle size and shape in addition to tuning particle dispersibility which would assist with material processing.

In this study, colloidal COF-300 crystallites are synthesized with rapid (15 minute–5 day) reaction times producing hydrated COF-300. This material is characterized *via* pair distribution function analysis to show their long-range ordering and crystallinity. A series of *para*-substituted benzoic acid catalysts are studied as the imine condensation catalyst. This series of *para*-substituted carboxylic acid catalysts offers fine-tuned control over acidity, which we initially hypothesized to affect final particle size *via* an effect on both imine condensation rates and acid/base interactions with amine-rich COF-300 colloid surfaces. Zeta potential experiments aimed at probing colloidal surface chemistry are consistent with positively charged ammonium benzoate surface species as a mechanism for colloidal stability. Additionally, ^1^H NMR model compound studies are used to elucidate the imine condensation *vs.* acid–base neutralization equilibria hypothesized to be responsible for the observed phenomena. Although we find that acidity of *para*-substituted benzoic acids affects time to particle nucleation, steric bulk of benzoic acids provides a more reliable way to tune particle size. These experiments will deepen our chemical understanding of colloidal 3D imine-linked COF nucleation and growth processes, enabling synthesis of 3D COFs with control of particle size.

## Results and discussion

### COF-300 colloid synthesis

**Scheme 1:**
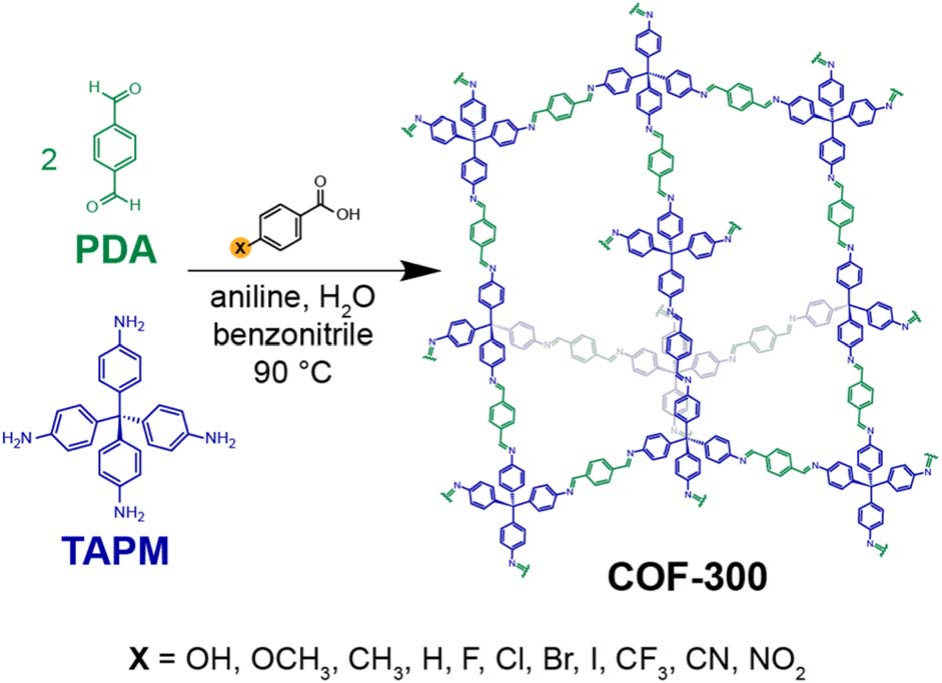
COF-300 colloid synthesis.

**Fig. 1:**
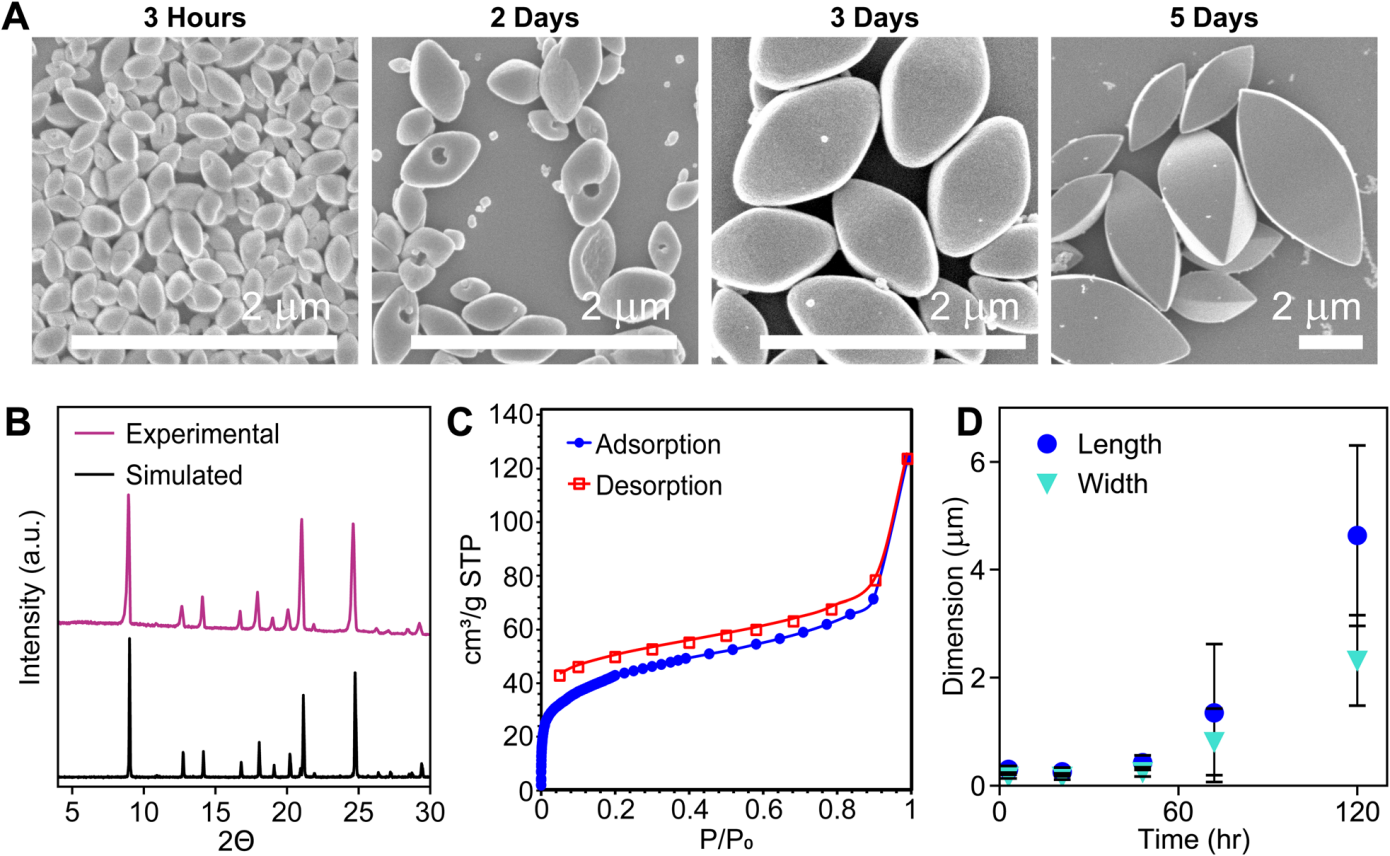
(A) SEM images of colloidal COF-300 (X = H) particle growth at 90 °C, (B) PXRD: experimental data (top) and simulated hydrated COF-300 pattern (bottom),^1^ (C) N_2_ adsorption isotherm, and (D) dimensions as determined *via* SEM measurements.

Briefly, colloidal COF-300 was synthesized *via* hot injection of a terephthaldehyde (PDA) solution in benzonitrile, to a solution of tetrakis(4-aminophenyl)methane (TAPM), aniline, water, and a substituted benzoic acid catalyst in benzonitrile (Scheme 1 and Fig. S1; see ESI† for detailed procedure). After 3 hours of reaction at 90 °C, a yellow colloid was observed, comprised of 400 nm diamondoid particles as observed *via* scanning electron microscopy (SEM) (Fig. 1A). These colloidal crystallites were purified by extraction using methanol and activated by supercritical CO_2_ drying for subsequent characterization by powder X-ray diffraction (PXRD), Fourier transform infrared spectroscopy (FTIR), and N_2_ porosimetry. The PXRD pattern of the COF exhibited sharp Bragg diffraction peaks, consistent with the hydrated structure of COF-300 (Fig. 1B) and reported powder patterns.^1^ FT-IR spectroscopy indicated successful formation of an imine bond *via* the appearance of a C=N imine bond stretch located at 1620.5 cm^−1^, accompanied by the disappearance of both the C–N stretch attributed to TAPM at 1271 cm^−1^ and C=O stretch from PDA at 1693 cm^−1^ (Fig. S2†).^38^ Analysis of the N_2_ adsorption isotherm provided a Brunauer–Emmett–Teller (BET) surface area of 150 m^2^ g^−1^ (Fig. 1C and S3†). These bulk characterization techniques collectively indicate that formation of COF-300 colloids under these conditions provide the target material with good quality.

The COF-300 reaction progress was monitored by taking reaction aliquots and characterizing the products *via* SEM performed on a Hitachi S4800 cFEG SEM. SEM imaging showed that COF-300 particles continued to grow over the span of 5 days, reaching dimensions of approximately 4.6 μm in length and 2.3 μm in width (Fig. 1A and D and S4†). However, as the particles grew, they began to precipitate, hindering the study of colloidal growth and their surface chemistry. As such, the COF-300 particles studied throughout the bulk of this manuscript are characterized after a reaction time of 48 hours, optimizing for reaction progress and colloidal stability.

### Pair distribution function analysis

**Fig. 2:**
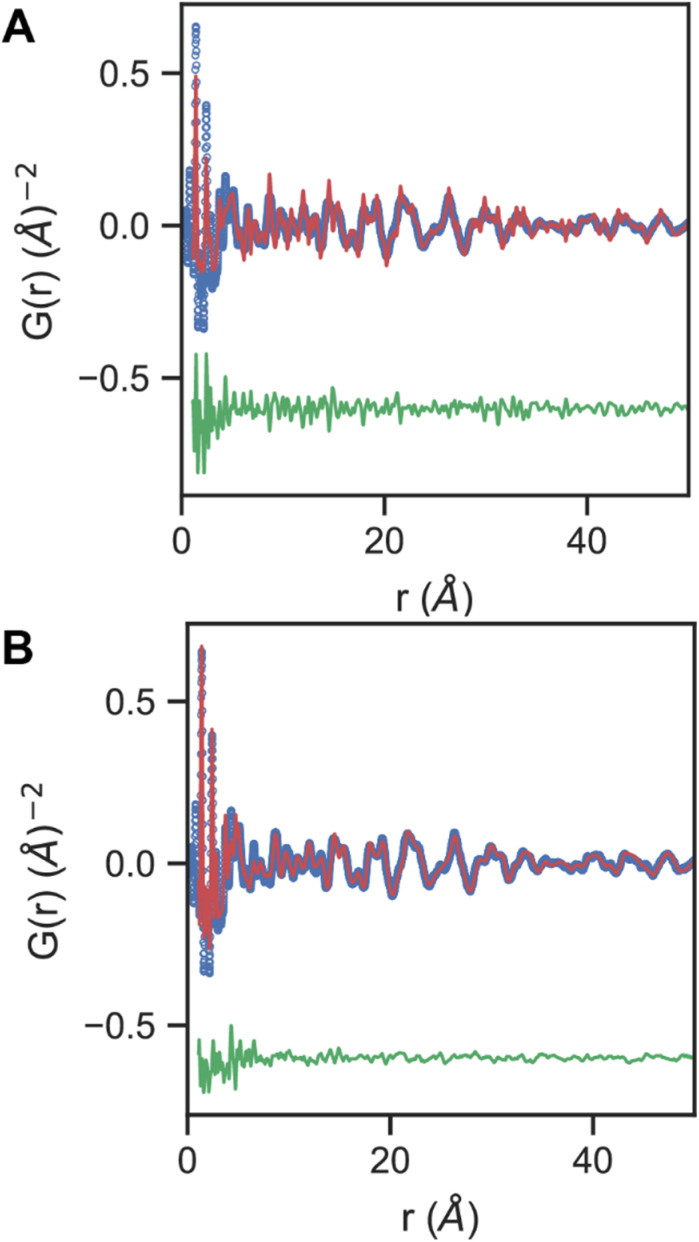
(A) Standard one phase crystallographic pair distribution function model (red), data (blue), and difference (green) above and (B) model with separate inter and intra monomer ADPs.

The structure of these COF-300 samples was examined by fitting of the real space Pair Distribution Function (PDF) from X-ray Total Scattering (Fig. 2). Research interest in porous and polymeric COFs has primarily focused on crystalline compounds as assessed by low angle Bragg diffraction peaks in powder XRD and single crystal XRD. However, recent studies that examine both Bragg and diffuse scattering have suggested significant local structural deviations from the average structure in these supramolecular materials.^39–41^ In one example, Terban and co-workers demonstrated that modeling of interlayer stacking disorder not captured in the average structure was necessary to account for the pair distribution functions.^41^ While deviations in the interlayer stacking of 2D COFs may be expected given the relatively weak dispersive forces responsible for the ordering of layers, the extent of disorder in 3D crystalline COFs is not well studied.

Here we show that attempts to fit crystalline 3D COF-300 PDFs using a standard crystallographic “Real Space Rietveld,” approach fails to properly account for the experimental PDFs for local and average real space distance ranges simultaneously. In particular, the local (low *r*) and average (high *r*) distance ranges refine very different values for the atomic displacement parameters (ADPs) needed to account for the line width of real space correlations (Fig. S29†). These deviations cannot be simply accounted for with correlated atomic motion factors.^42^ Instead, we adopt a modeling approach analogous to prior modeling of organic crystalline molecular materials, where both “intermolecular” and “intramolecular,” ADPs are separately refined for different real space pair distance ranges (See ESI†).^43,44^ Such models are known to account for deviations from the average structure that arise in crystalline molecular solids where deviations from intermolecular average distances have less of an energetic barrier than intramolecular average distances that arise from covalent bonds.^45^ Surprisingly, similar models are also consistent with these polymeric crystalline 3D COFs across a wide range of dimensions (length = 251–936 nm), suggesting that local structural deviations are far more energetically accessible in these porous supramolecular materials than conventional crystalline atomic solids.

### *Para*-substituted benzoic acid catalysts

In an attempt to further tune particle size, a series of *para*-substituted benzoic acid catalysts were screened (Table 1 and Fig. S5†). This fine-tuned control of acidity was hypothesized to tune the rate of imine condensation, resulting in variable colloidal particle sizes. Out of eleven *para*-substituted benzoic acids screened, five were readily soluble in benzonitrile at standard reaction conditions. Three initially insoluble ones (X = CF_3_, CN, Cl) did not fully solubilize at elevated temperatures until after the addition of water and aniline. Three additional benzoic acid derivatives (X = Br, I, NO_2_) never fully dissolved, and thus were not pursued further. Upon hot injection of PDA, one reaction (X = Cl) produced an aggregated, heterogeneous mixture. A primary benefit of colloidal COF synthesis is avoiding premature aggregation to increase crystallite size *via* error correction.^24,25^ Thus the *para*-substituted benzoic acids that did not produce colloidal products (X = Cl, Br, I, NO_2_) were not pursued further.

**Table 1:** *Para*-substituted benzoic acids studied and the stability of the resulting COF-300 colloids

X	Hammett parameter (*σ*)	Colloidally stable?
OH	−0.37	Yes
OCH_3_	−0.288	Yes
CH_3_	−0.17	Yes
H	0	Yes
F	0.062	Yes
Cl	0.227	No
Br	0.232	No
I	0.276	No
CF_3_	0.54	Yes
CN	0.66	Yes
NO_2_	0.778	No

The remaining benzoic acid derivatives, as shown in Table 1, produced colloidally stable COFs, as measured by FTIR and PXRD. Prior to characterization, the COF colloids were purified *via* successive precipitation, centrifugation, and resuspension cycles to remove unreacted monomers, excess benzoic acid, and benzonitrile solvent (see ESI†). The resulting COFs were dried under vacuum and isolated as solids before further characterization. FTIR measurements indicate successful formation of an imine bond linkage (Fig. S6–S11†). COF crystallinity was measured by a Siemens D5000 Diffractometer and a Rigaku MiniFlex-600, indicating formation of the hydrated COF-300 (Fig. S12 and S13†).^1,46^ Use of benzonitrile as the reaction solvent was crucial for both colloidal stability and crystallinity of the resulting materials.

**Fig. 3:**
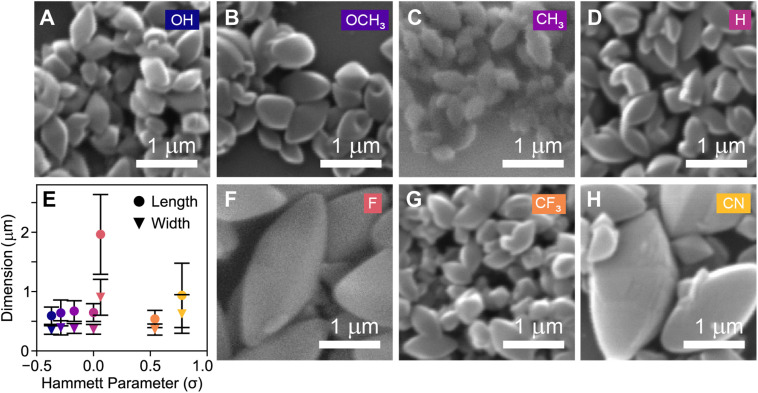
(A–D, F–H) SEM of COF-300 colloids synthesized at 90 °C for 48 hours made from different benzoic acids. (E) Average colloid length and width distribution data plotted *vs.* Hammett parameter.

To investigate the impact of these substituted benzoic acids on final particle size and morphology, SEM data was obtained on a FEI Quanta 200 ESEM. These SEM measurements demonstrate formation of well-faceted COF-300 crystallites (Fig. 3A–D, F–H) consistent with the morphology of COF-300 crystallites observed in the literature.^1^ An average of 250 particle lengths and widths were sized in ImageJ for each sample, to ensure representative sizing statistics (Fig. S14†). To assess the impact of *para*-substituted benzoic acid catalysts on particle morphology, the particle dimensions (length and width) were plotted against the Hammett parameter (*σ*) which is a measure of carboxylic acid strength (Fig. 3E). Interestingly, COF-300 colloids synthesized with 4-fluorobenzoic acid (X = F; *σ* = +0.062) and 4-cyanobenzoic acid (X = CN; *σ* = +0.66) were significantly larger than COF-300 particles synthesized with other substituted benzoic acids (X = OH, OCH_3_, CH_3_, H, CF_3_). These larger samples possessed average particle lengths and width of 1964 nm–936 nm and 904 nm–622 nm, respectively, compared with average lengths between 539 nm–646 nm and average widths between 353 nm–389 nm, respectively (Fig. 3 and S14†). Notably, the colloids synthesized with 4-fluorobenzoic acid and 4-cyanobenzoic acid possessed the largest size distributions.

Decreasing particle size dispersity is desirable for applications requiring monodisperse populations of particle sizes such as morphology-dependent adsorption studies.^33^ Among samples with similar particle dimensions (length and width), the particle size dispersity was studied to assess which benzoic acid derivatives resulted in highest monodispersity (Fig. S14†). The particle size dispersity was quantified as (*σ*/*d*) × 100 where *σ* is the standard deviation as measured by SEM sizing in ImageJ and *d* = particle dimension. A size dispersity was calculated for both the particle length and width. Notably, among the samples that were similarly-sized (average lengths between 539 nm–646 nm and average widths between 353 nm–389 nm), colloids synthesized with benzoic acid (X = H) possessed slightly narrower size distributions (*σ*/*d*_length_ = 23%, *σ*/*d*_width_ = 23%) than colloids synthesized with p-anisic acid (X = OCH_3_) (*σ*/*d*_length_ = 34%, *σ*/*d*_width_ = 31%). As observed by electron microscopy, carboxylic acid identity is observed to have a relatively minor impact on particle size and aspect ratio with the exception of the 4-cyanobenzoic acid and 4-fluorobenzoic acid derivatives. To rationalize the observed changes in particle size and size dispersities, we turned to a model study to understand the role of the carboxylic acid catalyst's role in imine condensation reactions.

### ^1^H NMR model compound studies

**Scheme 2:**
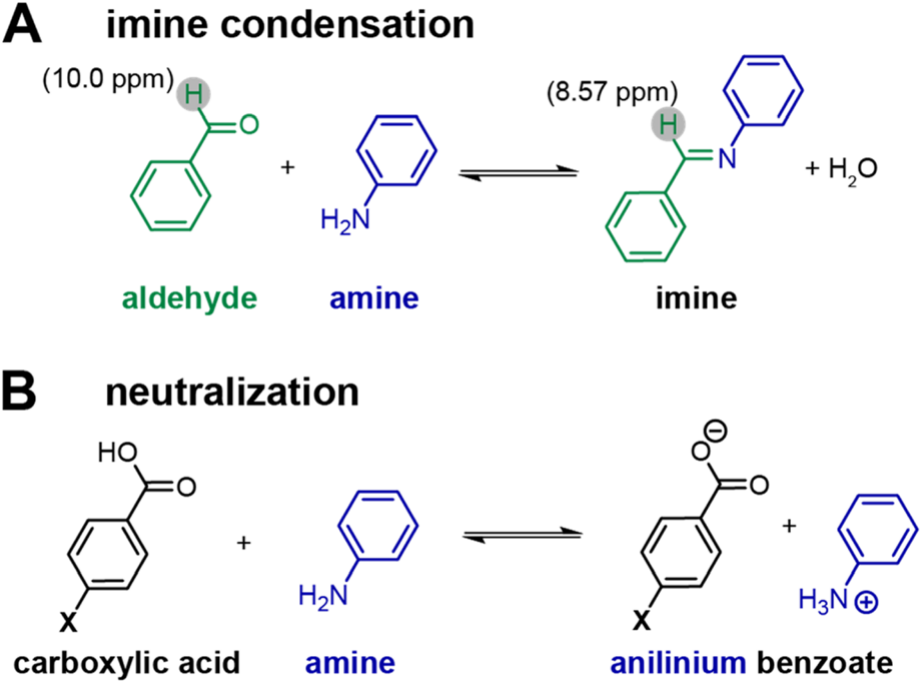
Carboxylic acid identity affects a series of equilibria: (A) imine condensation and (B) acid/base neutralization.

To probe the effect of *para*-substituted benzoic acids on the reaction progress of reversible reactions relevant for COF formation, model compound experiments were performed using aniline (a monofunctional amine) and benzaldehyde (a monofunctional aldehyde) in place of the multifunctional TAPM and PDA COF-300 monomers. ^1^H NMR experiments were set up in acetonitrile-d3 at a sixfold dilution compared to reaction conditions, in order to achieve good solubility of all compounds at room temperature. ^1^H NMR distinguishes between the aldehyde and imine protons that appear at 10.0 ppm and 8.57 ppm, respectively (Scheme 2).

**Fig. 4:**
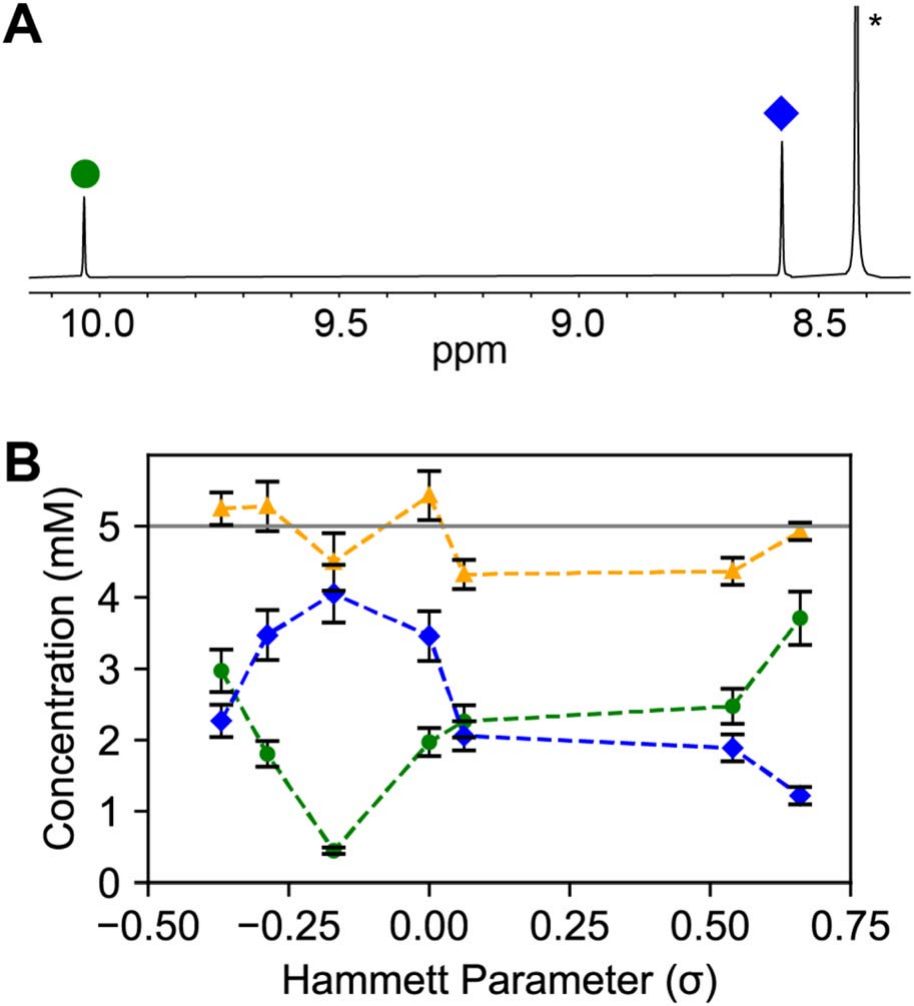
(A) ^1^H NMR of model compound study displays the ratio of imine (blue diamonds) *vs.* aldehyde (green circles) quantified relative to a 1,4-dinitrobenzene internal standard (*), (B) the relative concentrations of imine (blue diamonds) to aldehyde (green circles) changes as a function of *para*-substituted benzoic acid catalyst acidity as quantified *via* Hammett parameter. The total concentration of imine + aldehyde (yellow triangles) is plotted relative to the expected total concentration (grey line).

Imine and aldehyde concentrations are quantified *via* integration relative to a 1,4-dinitrobenzene internal standard (1.25 M). As the *para*-substituted benzoic acid becomes more acidic, an acid–base neutralization reaction between the carboxylic acid and the amine will occur, decreasing the amount of amine available and shifting the imine condensation equilibrium towards the reactants (Scheme 2). Thus, we observe that more acidic benzoic acids result in a higher benzaldehyde to imine ratio (Fig. 4). This was true for all benzoic acids tested with the exception of the 4-hydroxybenzoic acid which had a much lower imine concentration than expected.

**Fig. 5:**
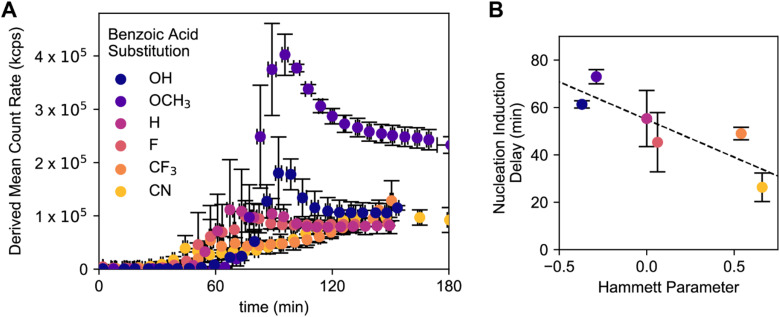
Effect of different benzoic acids on (A) derived mean count rate during synthesis as measured *via in situ* DLS and (B) nucleation induction delay as a function of Hammett parameter. Error bars come from experiments performed in triplicate.

We rationalize our results by contrasting this model compound study with the actual COF synthesis, where crystallization is a driving force that consumes aldehyde and disfavors depolymerization.^47^ Any initial bias towards benzaldehyde for more acidic benzoic acids is hypothesized to lead to faster productive imine–condensation reactions. We sought to probe this effect further by looking at nucleation induction delay times (Fig. 5B).

### *In situ* dynamic light scattering studies

Having observed the imine condensation equilibrium position *via* model compound studies, we sought to study the effects of benzoic acid derivative on nucleation induction delay times. *In situ* dynamic light scattering (DLS) experiments were performed to probe the time to colloid nucleation. All syntheses were performed under standard reaction conditions inside a temperature-controlled Malvern Zetasizer Pro. DLS measurements are attempted at approximately five minute intervals and measured for approximately one hour past the initial observation of light scattering. At the beginning of the reaction, the small molecules present in the reaction mixture do not scatter light.^48,49^ However, after colloid nucleation, the derived mean count rate increases and scattering objects of measurable sizes are detected. These experiments could not be performed for the entire duration of the COF reactions (48 hours), as a high reaction yield of COF-300 colloid results in high turbidity that can produce multiple scattering events.

Using the series of substituted benzoic acids, different times to nucleation are observed, as measured by an increase in derived mean scattering count rate (Fig. 5A, B and S15†). We observe that 4-cyanobenzoic acid, the most acidic benzoic acid, results in the shortest time to nucleation, while 4-methoxybenzoic acid results in the longest time to nucleation. There appears to be a negative correlation between Hammett parameter and nucleation induction delay, although several derivatives studied appear to be within error.

Interpretation of the *in situ* DLS studies brought up several additional questions. Although we hypothesized that COF-300 nucleation induction delay would track with the Hammett parameter of each benzoic acid, there appeared to be no correlation between nucleation induction delay times and final particle sizes as measured *via* SEM. Based on these results, we concluded that the final particle size is not solely linked to the carboxylic acid catalyst's acidity and activation of imine condensation.

### Surface chemistry studies

**Scheme 3:**
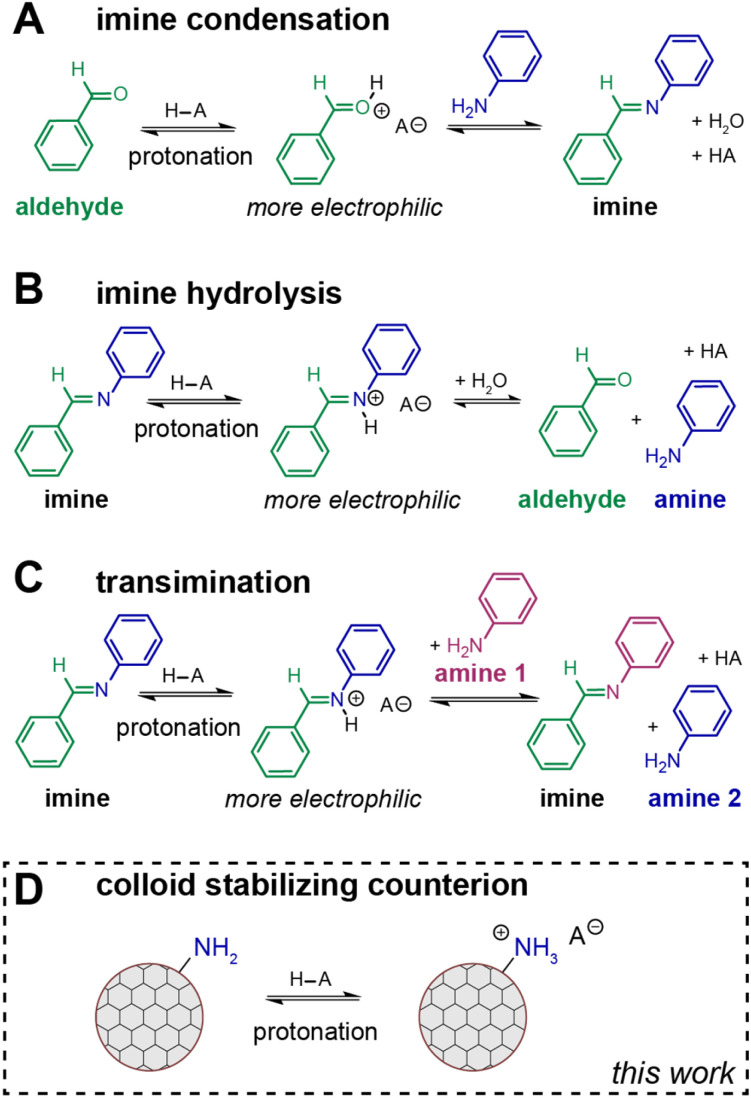
Acid plays multiple roles in imine-linked COF colloid synthesis including (A) acid-catalyzed imine condensation, (B) acid-catalyzed imine hydrolysis, (C) acid-catalyzed transimination, and (D) a colloid stabilizing counterion *via* formation of an acid–base adduct.

In addition to imine-formation and transimination kinetics, we hypothesized that the benzoic acid catalyst is responsible for colloid stabilization *via* acid–base chemistry (Scheme 3). We observe this through a series of experiments and observations. Traditionally in the COF colloid literature, upon completion of synthesis, COF colloids are flocculated by the addition of saturated sodium chloride (brine).^22,26^ The flocculated COF solids are collected in a teabag and purified *via* methanol Soxhlet extraction. The Soxhlet removes unreacted monomers and small oligomers from the reaction without checking the resulting material's colloidal stability. In this study, we sought to purify our smallest size of COF-300 colloids synthesized with benzoic acid and heated for 15 minutes at 90 °C *via* centrifugation, where the as-synthesized colloids were purified *via* addition of a nonpolar antisolvent (hexane) followed by centrifugation, decanting the supernatant containing unreacted monomers and small oligomers. Subsequent cleaning procedures used acetonitrile/toluene as the solvent/antisolvent (See ESI†). Interestingly, after three rounds of centrifugation and resuspension, the purified colloid was no longer colloidally stable in acetonitrile. At this point, GC/MS analysis of the supernatant from each round of centrifugation and FTIR of the isolated COF solids indicate full removal of benzoic acid from the COF-300 material (Fig. S16, S6–S11†). Benzoic acid was subsequently titrated back into the purified COF sample, resulting in resuspension of the colloids in acetonitrile.

**Fig. 6:**
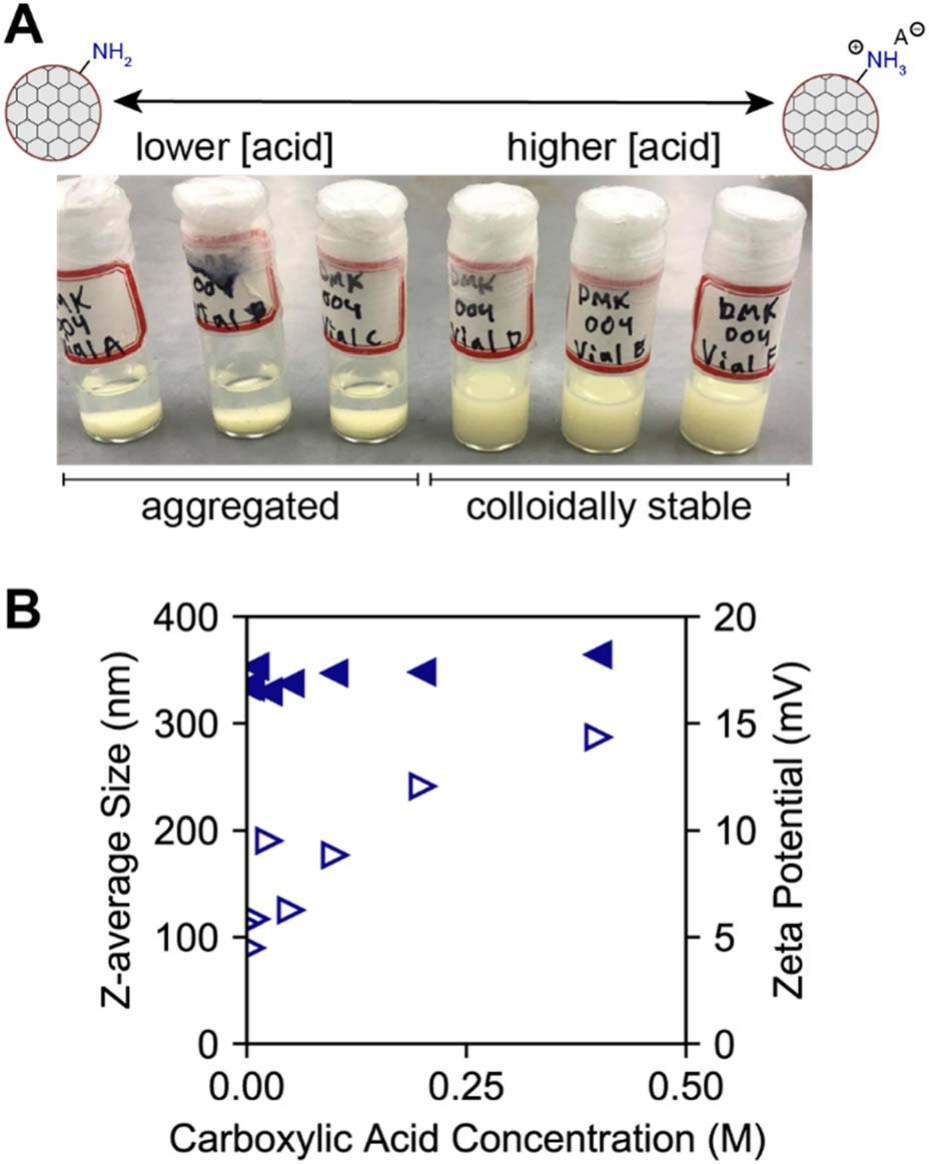
(A) COF-300 colloids that have been fully purified *via* centrifugation are colloidally stable in acetonitrile only in the presence of added benzoic acid. (B) Z-average size (nm) (represented as filled triangles) and zeta potential (mV) (represented as unfilled triangles) of COF-300 colloids purified *via* centrifugation and resuspended in benzonitrile as a function of carboxylic acid concentration.

To quantify the effect of benzoic acid concentration on COF-300 colloidal stability, DLS experiments and zeta potential measurements were performed. Visibly, a threshold concentration of 0.05 M benzoic acid in acetonitrile is observed to result in colloidal stability (Fig. 6A, S17–S19†). At 0.025 M benzoic acid in acetonitrile, particle sedimentation is observed. Aggregation is observed *via* the shifting intensity size distribution and corresponding correlograms (Fig. S20–S22†). Interestingly, in benzonitrile, the COF-300 particles remain colloidally stable over a range of benzoic acid concentrations even after full purification (Fig. 6B). This may be due to a difference in solvent dielectric coefficients (at 293.16 K, benzonitrile: *ε* = 25.66; acetonitrile: *ε* = 36.60)^50^ with acetonitrile stabilizing charged ammonium benzoate species. To probe the interaction of benzoic acid with the COF colloid surface, zeta potential experiments were performed in slow field reversal mode to accommodate slower particle movement in organic solvents in response to the applied electric field. Over a range of 0 to 0.4 M benzoic acid in benzonitrile, the measured zeta potential increases from +5.6 mV to +14.35 mV. This indicates increasing positive charge on the surface of the colloid with increasing carboxylic acid concentration, which we attribute to increasing surface amine protonation. Thus, we conclude that the interaction of benzoic acid with the COF surface is an important component of colloidal stability. Benzoic acid derivative solubility effects (Table S3†) may also be important and must be studied in future work.

Seeking further evidence of the interaction of benzoic acid with the COF particle surfaces, we performed diffusion ordered spectroscopy (DOSY). We hypothesized that we would observe a decrease in the experimental diffusion constant (*D*) from benzoic acid's ^1^H NMR signals, attributable to slowed diffusion *via* interaction with a slow-diffusing COF colloid surface. DOSY experiments were performed on a 400 MHz Bruker Avance III HD 400 MHz spectrometer. See ESI† for sample preparation and experimental details. The extracted diffusion constant for free benzoic acid (2.007 × 10^−9^ m^2^ s^−1^) was compared to a benzoic acid in the presence of purified COF-300 colloid (1.981 × 10^−9^ m^2^ s^−1^) in acetonitrile-d3. In accordance with the Stokes–Einstein equation, *D* is inversely correlated with the solute's hydrodynamic radius. The extremely minor change in diffusion constant observed is not fully conclusive and is likely within error of the experiment. It is likely that the lability of the acid–base neutralization reaction prevents observation of a larger decrease in diffusion constant.

**Fig. 7:**
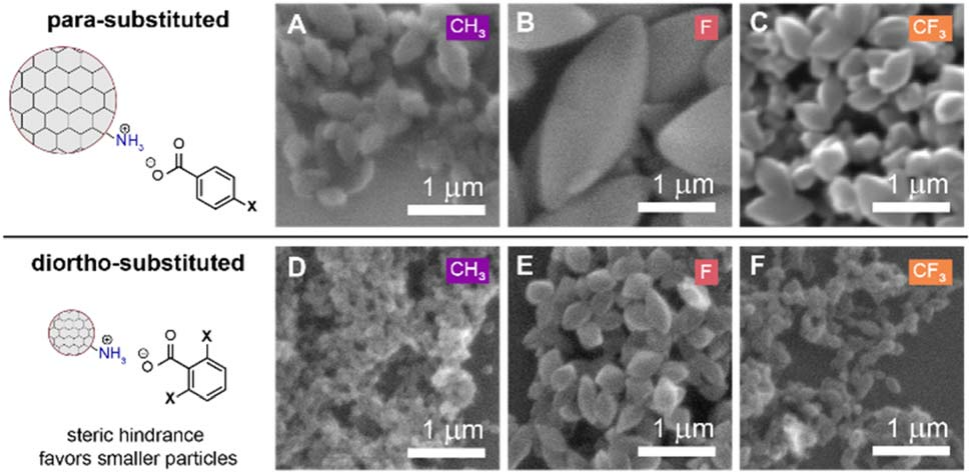
SEM images comparing COF-300 particle sizes as synthesized with (A–C) *para*-substituted benzoic acids (X = CH_3_, F, CF_3_) *vs.* particles synthesized with (D–F) diortho-substituted benzoic acids (X = CH_3_, F, CF_3_).

Based on our new model of colloidal 3D imine-linked COF surface chemistry featuring acid/base chemistry (Scheme 3D), we predicted that sterically hindered carboxylic acids would result in smaller particle sizes. Smaller particles possess higher surface curvature which can more easily accommodate steric bulk near the reactive carboxylic acid functional group. This phenomenon has been proposed for other colloidal particle systems such as CdSe magic size clusters showing higher surface ligand densities than larger CdSe nanoparticles.^51^ To probe this hypothesis, colloidal COF-300 particles were synthesized using our standard reaction conditions (90 °C, 48 hours) and 2,6-dimethylbenzoic acid, 2,6-difluorobenzoic acid, or 2,6-bis(trifluoromethyl)benzoic acid. Upon purification of the resulting products, we confirmed successful imine condensation and crystallization *via* FTIR and XRD (Fig. S23–S26†). Subsequent SEM imaging showed that sterically bulky diortho-substituted benzoic acids across all tested substitutions (X = CH_3_, F, CF_3_) resulted in smaller particle sizes compared to their *para*-substituted counterparts (average length = 158 nm (2,6-dimethylbenzoic acid) *vs.* 672 nm (4-methylbenzoic acid); 436 nm (2,6-difluorobenzoic acid) *vs.* 1964 nm (4-fluorobenzoic acid); 244 nm (2,6-bis(trifluoromethyl)benzoic acid) *vs.* 539 nm (4-trifluoromethylbenzoic acid)) (Fig. 7 and S27†). Further investigation is required to more thoroughly explore the impact that sterically bulky carboxylic acids have on particle nucleation kinetics and final particle sizes.

## Conclusions

As a result of our evidence for the interaction of benzoic acid with the surface of the COF colloid being responsible for colloidal stability in high dielectric solvents, we were able to leverage our findings to rationally control particle size. Contrary to our hypothesis, looking at electronic effects of *para*-substituted benzoic acids, we observe the largest particle sizes with 4-fluorobenzoic acid and 4-cyanobenzoic acid which differ significantly in terms of acidity as measured *via* influence on the imine/aldehyde equilibrium in our model compound studies and nucleation induction delay times as measured by *in situ* DLS. However, the use of sterically hindered diortho-substituted carboxylic acid catalysts was demonstrated to result in smaller particle sizes across all substitutions tested. These results taken together provide insight into 3D imine-linked COF colloid surface chemistry. Previously, researchers have studied a variety of organic and Lewis acids' role in imine condensation, imine hydrolysis, and transimination reactions (Scheme 3A–C).^52–54^ Here we propose that the carboxylic acid catalyst also serves as a colloid stabilizing counterion *via* surface amine protonation (Scheme 3D). We also note that imine protonation could similarly provide colloidal stabilization, as iminium protonation has previously been invoked in macrocycle self-assembly.^55^ The full potential of understanding COF colloid surface chemistry lies in accessing COF crystallites with different sizes and morphologies. Several examples in the colloidal and non-colloidal porous framework literature demonstrate size tunability with the addition of molecules that can interact with the colloid surfaces (*e.g.* modulators)^1,21,33,49,56^ or the addition of different amounts of acetic acid.^37^

As identification and optimization of functional COF monomers reaches maturity, the next frontier of materials property optimization will occur in particle size and shape control. To date, most property studies focus on tuning the chemical affinity and pore size *via* monomer choice. Because dynamic crystallization conditions have thus far proven to be challenging, most research has optimized for the largest possible crystalline domain sizes. With the introduction of robust, facile COF syntheses enabled by colloidal synthesis, further optimization of applications centered on COF crystallite morphology become possible. Small COF particle sizes enabled by sterically hindered carboxylic acid catalysts possess increased surface to volume ratios, and the chemistry of unreacted monomer functional groups, or polymer end groups, will lead to enhanced materials performance.

## Author contributions

W. J., D. M. K., B. M. P., K. J. C., A. C. C., A. J. C., A. F. M, S. M. O., D. V. T, and L. S. H. performed and interpreted COF growth and characterization experiments. W. J., B. M. P., A. C. C., K. J. C., and L. S. H. performed and interpreted SEM experiments. D. M. K. and L. S. H. performed and interpreted NMR experiments. B. M. P., S. M. O, A. F. M. and L. S. H. performed and interpreted DLS experiments. A. J. C., D. V. T., and L. S. H. performed and interpreted GC/MS experiments. M. H. and M. W. G. performed and interpreted PDF analysis. All authors wrote and revised the manuscript.

## Conflicts of interest

The authors declare no conflicts of interest.

## Supplementary Material

RA-013-D3RA02202A-s001
