# Supplementary material for: COF-300 synthesis and colloidal stabilization with substituted benzoic acids
Source: RSC Adv. 2023 May 12;13(21):14484–93. doi: 10.1039/d3ra02202a (PMC10176042; doi:10.1039/d3ra02202a)
Supplement: RA-013-D3RA02202A-s001 [file RA-013-D3RA02202A-s001.pdf]

Electronic Supplementary Material (ESI) for RSC Advances.
This journal is © The Royal Society of Chemistry 2023

COF-300 Synthesis and Colloidal Stabilization with Substituted Benzoic Acid Catalysts

Woojung Ji, [1] Dean M. Kim,[2] Brendan M. Posson,[2] Kyla J. Carlson, [3] Alison C. Chew, [2,3] Alyssa J. Chew,[2] Meherin Hossain, [4] Alexis F. Mojica, [2] Sachi M. Ottoes,[2] Donna V. Tran,[2] Matthew W. Greenberg,[4] and Leslie S. Hamachi[2,]*

[1]*Department of Chemistry, University of Michigan,*
Ann Arbor, MI, 48109, USA
[2]*Department of Chemistry and Biochemistry, California Polytechnic State University,*
San Luis Obispo, CA, 93407, USA
[3]*Department of Materials Engineering, California Polytechnic State University,*
San Luis Obispo, CA, 93407, USA
[4]*Department of Chemistry and Biochemistry, Bard College,*
Annandale-on-Hudson, NY, 12504, USA

Supplementary Information

**Correspondence Address**
Professor Leslie S. Hamachi<br>Department of Chemistry and Biochemistry<br>California Polytechnic State University<br>San Luis Obispo, CA, 93407 (USA)<br>hamachi@calpoly.edu

Table of Contents

A. Materials and Instrumentation S-2
B. Synthetic Procedures S-6
C. Characterization Tables and Figures S-11
D. Pair Distribution Function Analysis S-31
E. ^{1}H NMR Spectra S-35
F. References S-46

A. Materials and Instrumentation.

Materials. Reagents were purchased in reagent grade from commercial suppliers and used without further purification, unless otherwise described. Tetrakis-4-aminophenyl methane (TAPM, ≥95%) was purchased from Accela Chembio Inc. Benzonitrile (>99.0%), 4-hydroxybenzoic acid (>99%), 4-bromobenzoic acid (>98.0%), 4-iodobenzoic acid (>98.0%), 4-trifluoromethylbenzoic acid (>98%), and 4-cyanobenzoic acid (>98.0%) were purchased from TCI Chemicals. Terephthaldehyde (PDA, 98%), p-toluic acid (98%), 4-nitrobenzoic acid (>99%), and p-anisic acid (≥98%) were purchased from ACROS organics. Benzaldehyde (≥99%) was purchased from Millipore Sigma. Aniline (≥99%), benzoic acid (≥99%), and 4-chlorobenzoic acid (≥98%), and 4-fluorobenzoic acid (98%), were purchased from Alfa Aesar. Acetonitrile-d3 (99.5 atom % D) was purchased from Thermo Scientific. Acetonitrile (Certified ACS), hexanes (Technical Grade) and toluene (Laboratory Grade) were purchased from Fisher Chemical.

Instruments.

Fourier Transform Infrared spectra (FTIR) were recorded using a Nicolet iS10 FT-IR spectrometer equipped with a diamond ATR.

Proton nuclear magnetic resonance (^{1}H NMR) spectra were recorded on a Bruker-300 UltraShield with an Avance III HD spectrometer. Diffusion ordered spectroscopy (DOSY) experiments were performed on a Bruker Avance III HD 400 MHz spectrometer.

Gas Chromatography/Mass Spectrometry was performed on an Agilent 6890 N Network GC System with an Agilent 5973Network Mass Selective Detector.

Dynamic Light Scattering (DLS) and Zeta potential measurements were performed on a Malvern Zetasizer Pro.

Scanning Electron Microscopy (SEM) was performed on a FEI Quanta 200 ESEM. Prior to characterization, 50 μL of COF-300 colloid is diluted in 950 μL of acetonitrile, drop cast onto a silicon wafer, and allowed to dry overnight in a fume hood. Samples are mounted on aluminum pucks (Ted Pella) with double-sided carbon tape prior to sputter coating with gold using a Cressington Model 108 Auto Sputter Coater. Micrographs were taken with 30 kV accelerating voltage and a spot size of 4.0.

SEM images of COF-300 colloids isolated at different time points (Figure 1A) were taken on a Hitachi S4800 cFEG SEM. The samples were coated with 8 nm of Osmium using SPI OPC-60A osmium plasma coater.

ImageJ software is used to measure the length and width of the COF-300 particles under all synthetic conditions. Approximately 100-350 individual particles were measured for each reaction condition where micrographs were taken.

X-ray diffraction (XRD) was performed on a Siemens D5000 X-ray Diffractometer equipped with a scintillation detector. Measurements were performed with a divergence slit of 0.1 mm, anti-scatter slit of 0.1 mm, and a detector slit of 0.6 mm at a scanning rate of 5°/min.

Powder X-ray diffraction (PXRD) patterns (Figure 1) were obtained at room temperature on a STOESTADIMP powder diffractometer equipped with an asymmetric curved Germanium monochromator (CuKα1 radiation, λ = 1.54056 Å) and one-dimensional silicon strip detector (MYTHEN2 1K from DECTRIS). The line focused Cu X-ray tube was operated at 40 kV and 40 mA. The as-obtained powder samples were sandwiched between two acetate foils (polymer sample

with neither Bragg reflections nor broad peaks above 10° 2θ) mounted in flat plates with a disc S-3 opening diameter of 8 mm and measured in transmission geometry in a rotating holder. The patterns were recorded in the 2θ range of 0–35° for an overall exposure time of 24 min. The instrument was calibrated against a NIST Silicon standard (640d) prior to the measurement.

X-Ray Diffraction (XRD) measurements were performed on a Rigaku MiniFlex-600. This instrument was equipped with a 0.625° divergence slit and a 0.3 mm standard detector slit. Data was collected over a 2θ range of 4-30° and a scan rate of 4°/minute.

PXRD was performed on a Panalytical Empyrean Powder Diffractometer equipped with a Cu X-ray tube operated at 45 kV and 40 mA with a PIXcel 1D detector. Data was collected over a 2θ range of 5-30° with a 10 minute exposure time.

For samples in Figure 1, supercritical CO_2 drying was performed on Leica EM CPD 300. Prior to the supercritical drying process, samples were placed in tea bags (ETS Drawstring Tea Filters, sold by English Tea Store) while wet. The tea bags containing the samples were then placed in the drying chamber. The drying chamber was first sealed, cooled, and filled with liquid CO_2, and after 2 min, the samples were vented quickly. This fill-vent cycle was repeated 99 times, after which the temperature was raised to 40 °C resulting in a chamber pressure of around 1300 psi, which is well above the critical point of CO_2. The chamber was held above the critical point for 5 min, after which the CO_2 source was turned off, and the pressure was released over a period of 5 min.

Gas adsorption isotherms were conducted on a Micromeritics ASAP 2420 Accelerated Surface Area and Porosity Analyzer using 15–50 mg samples in dried and tared analysis tubes equipped with filler rods and capped with a Transeal. Samples were heated to 40 °C at a rate of 1

°C/min and evacuated at 40 °C for 20 min, then heated to 100 °C at a rate of 1 °C/min heat and evacuated at 100 °C for 18 h. After degassing, each tube was weighed again to determine the mass of the activated sample and transferred to the analysis port of the instrument. UHP-grade (99.999% purity) N_2 was used for all adsorption measurements. N_2 isotherms were generated by incremental exposure to nitrogen up to 760 mmHg (1 atm) in a liquid nitrogen (77 K) bath. Oil-free vacuum pumps and oil-free pressure regulators were used for all measurements. Brunauer-Emmett-Teller (BET) surface areas were calculated from the linear region of the N_2 isotherm at 77 K within the pressure range P/P0 of 0.10–0.20.

X-ray total scattering measurements were taken at The National Synchrotron Light Source II XPD beamline (XPD, 28-ID2) at Brookhaven National Laboratory. COF powder samples are loaded into 1.5 mm borosilicate glass tubes. Diffraction images were collected at room temperature with an X-ray energy of 67.178 keV (λ = 0.18456 Å) using a large area 2D PerkinElmer detector in transmission geometry. The experimental sample-to-detector distance was set at 242.61 mm. The detector 2θ range range and experimental geometry are calibrated by measuring crystalline nickel powder standard directly prior to measurements of the COF, with the experimental geometry parameters refined using the pyFAI program.[1]

Pair Distribution Function Analysis and Data Modeling was performed in xPDFsuite. The program xPDFsuite with PDFgetX3 is used to subtract the background scattering and normalize experimental $I(Q)$ to obtain the reduced total scattering structure function $F(Q)$, and Fourier transformed to obtain the pair distribution function (PDF).[2] The Q_{max} for generation of the $G(r)$ was set at 22 Å^{-1}. Least squares refinement to fit models of $G(r)$ to experimental data. were performed in PDFgui.[3]

B. Synthetic Procedures

Scheme S1

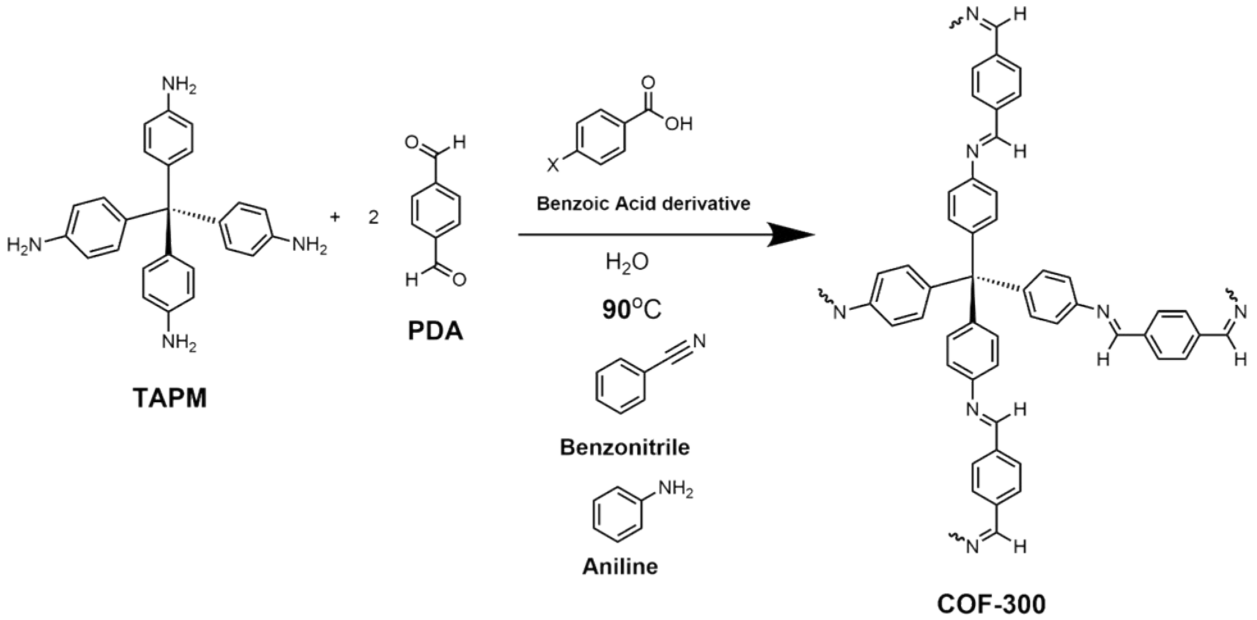

COF-300 Colloid Synthesis

A 20 mL glass vial is loaded with 0.019 g (0.05 mmol) of TAPM, a para-substituted benzoic acid derivative (1.9 mmol, see **Table S1**) and 5.0 mL of benzonitrile. This reaction mixture was sonicated for 15 seconds and subsequently heated to 90 °C. Upon dissolution at elevated temperatures, 0.08 mL of 0.7 M aniline in benzonitrile (0.056 mmol) and 0.12 mL of water (6.67 mmol) are added to the reaction mixture. A separate 4 mL vial is loaded with 0.012 g of PDA (0.09 mmol) dissolved in 1.0 mL of benzonitrile. The PDA solution is added to the TAPM solution and held at 90 °C for the desired time (15 minutes to 48 hours). After completion, the reaction mixture is allowed to sit at room temperature for 12 hours before further characterization.

Table S1. Properties and amounts of para-substituted benzoic acids studied and the stability of the resulting COF-300 colloids.

X	MW of Benzoic Acid (g/mol)	mmols used	Reagent used (mg)	Hammett Parameter (σ)	Colloidally Stable?
OH	138.12	1.867	257.87	-0.37	Yes
OCH_3	152.15	1.867	284.06	-0.288	Yes
CH_3	136.15	1.867	254.19	-0.17	Yes
H	122.12	1.867	228.00	0	Yes
F	140.11	1.867	261.59	0.062	Yes
Cl	156.57	1.867	292.32	0.227	No
Br	201.02	1.867	375.30	0.232	No
I	248.02	1.867	463.05	0.276	No
CF_3	190.12	1.867	354.95	0.54	Yes
CN	147.13	1.867	274.69	0.66	Yes
NO_2	167.12	1.867	312.01	0.778	No

COF Purification Process

After sitting at room temperature for 12 hours, the reaction mixture was transferred to a 50 mL centrifuge tube. Approximately 24 mL of hexane was added to the reaction mixture. The centrifuge tube was inverted several times and sonicated for 10 seconds to ensure full mixing prior to centrifugation at 3000 rpm for 15 minutes. Subsequently, the supernatant was decanted and the remaining solids were resuspended in 6 mL of acetonitrile. After resuspension, the mixture was sonicated for 15 seconds prior to the addition of 12 mL of toluene. The suspension was centrifuged at 3000 rpm for 15 minutes and the supernatant was decanted. The resuspension in acetonitrile and centrifugation process was repeated once to ensure full purification of the COF-300 sample.

The cleaned COF-300 samples were then dried under vacuum for 24 hours to remove residual solvent. The isolated COF-300 solids were subsequently characterized by FTIR and XRD.

GC/MS Characterization of Supernatant

COF-300 was synthesized and purified via the centrifugation purification process described above. To prepare each sample for GC/MS, each supernatant was filtered through a 0.45 µm PTFE syringe filter. The graph shows that the cleaning procedure is effective, as reactant and cleaning solvents peaks are absent by the 3rd supernatant.

***In situ* DLS Experiments**

The COF-300 colloid synthesis is performed as described above. Upon addition of all reagents, a 1 mL reaction aliquot is transferred to a cuvette pre-heated to 90 °C in the Malvern Zetasizer Pro Blue for *in situ* DLS measurements. DLS measurements were performed every 5 minutes. Nucleation induction delay was determined based on the time to obtain the first measurable particle size. Measurements were taken until the particle size measurements reached an asymptotic value or the instrument recorded an error due to high sample concentration.

^{1}H NMR Experiments

An internal standard solution with 1,4-dinitrobenzene was prepared by dissolving 0.120 mmol (20.2 mg) of 1,4-dinitrobenzene in 16.0 mL of acetonitrile-d3. A model compound stock solution was prepared by mixing 0.149 mmol (13.9 mg) of aniline, 0.105 mmol of benzaldehyde (11.1 mg), 3.88 mmol of water (69.9 mg), and 3.5 mL of the internal standard solution. After preparation of both solutions, 0.3 mL of the reagent stock solution was diluted sixfold with 1.5 mL of the internal standard solution and 0.093 mmol of a para-substituted benzoic acid derivative (see **Table S2**) was dissolved in the mixture. A 0.6 mL aliquot of this diluted mixture was transferred

to an NMR tube for ^{1}H NMR characterization. Data was obtained at room temperature, approximately 1 hour after sample preparation.

Table S2. Amount of para-substituted benzoic acid used for the model compound ^{1}H NMR characterization.

Reagent	MW (g/mol)	mmols used	Reagent used (mg)
4-Hydroxybenzoic acid	138.12	0.093	12.9
Anisic acid	152.15	0.093	14.2
4-Methylbenzoic acid	136.15	0.093	12.7
Benzoic acid	122.12	0.093	11.4
4-Fluorobenzoic acid	140.11	0.093	13.1
4-(Trifluoromethyl)benzoic acid	190.12	0.093	17.7
4-Cyanobenzoic acid	147.13	0.093	13.7

DOSY NMR Experiments

A benzoic acid stock solution was prepared by dissolving 0.408 mmol (49.8 mg) of benzoic acid and 0.015 mmol (2.5 mg) of 1,4-dinitrobenzene in 2 mL of acetonitrile-d3. A 0.6 mL aliquot of this solution was transferred to an NMR tube for DOSY analysis. DOSY measurements were performed with a longitudinal eddy current delay bipolar gradient pulse sequence (ledbpgp2s) from Bruker. The gradient strength (GPZ6) was varied linearly from 5 to 95% of the probe's maximum value in 16 steps. For the acquisition parameters, the diffusion time (Δ) and gradient pulse duration (δ) were optimized to 0.0399 s and 800 µs respectively. The COF-300 sample with benzoic acid was prepared with a cleaned COF-300 using the purification process described above. After drying the cleaned colloid, the sample was resuspended in 6 mL of acetonitrile. A 0.5 mL

aliquot of this suspension was transferred and mixed with 1 mL of toluene to be centrifuged at 3000 rpm for 15 minutes. After decanting, the resuspension and centrifugation process were repeated using acetonitrile-d3 instead as the solvent. The cleaned COF-300 was resuspended in 1 mL of the benzoic acid stock solution in acetonitrile-d3 and a 0.6 mL aliquot was removed for DOSY analysis using the same acquisition parameters as the above.

C. Characterization Tables and Figures

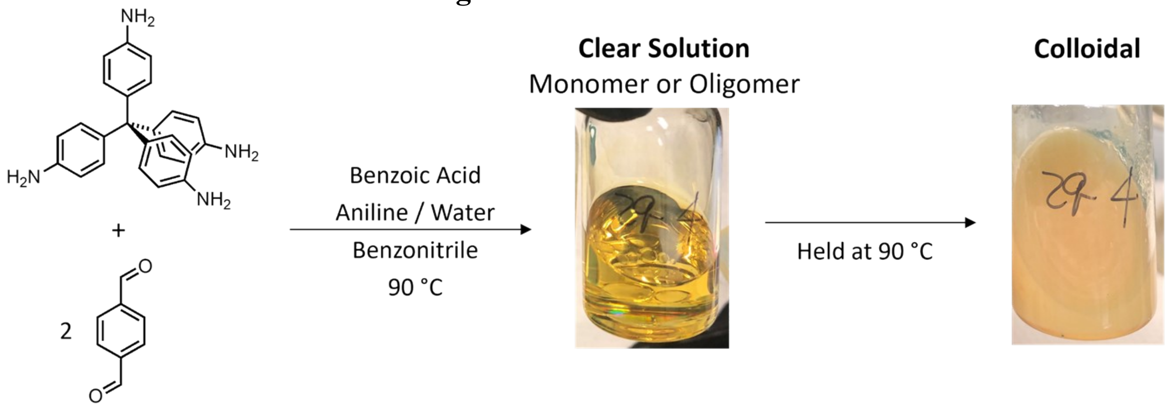

Figure S1. Visual representation of colloidal COF-300 formation

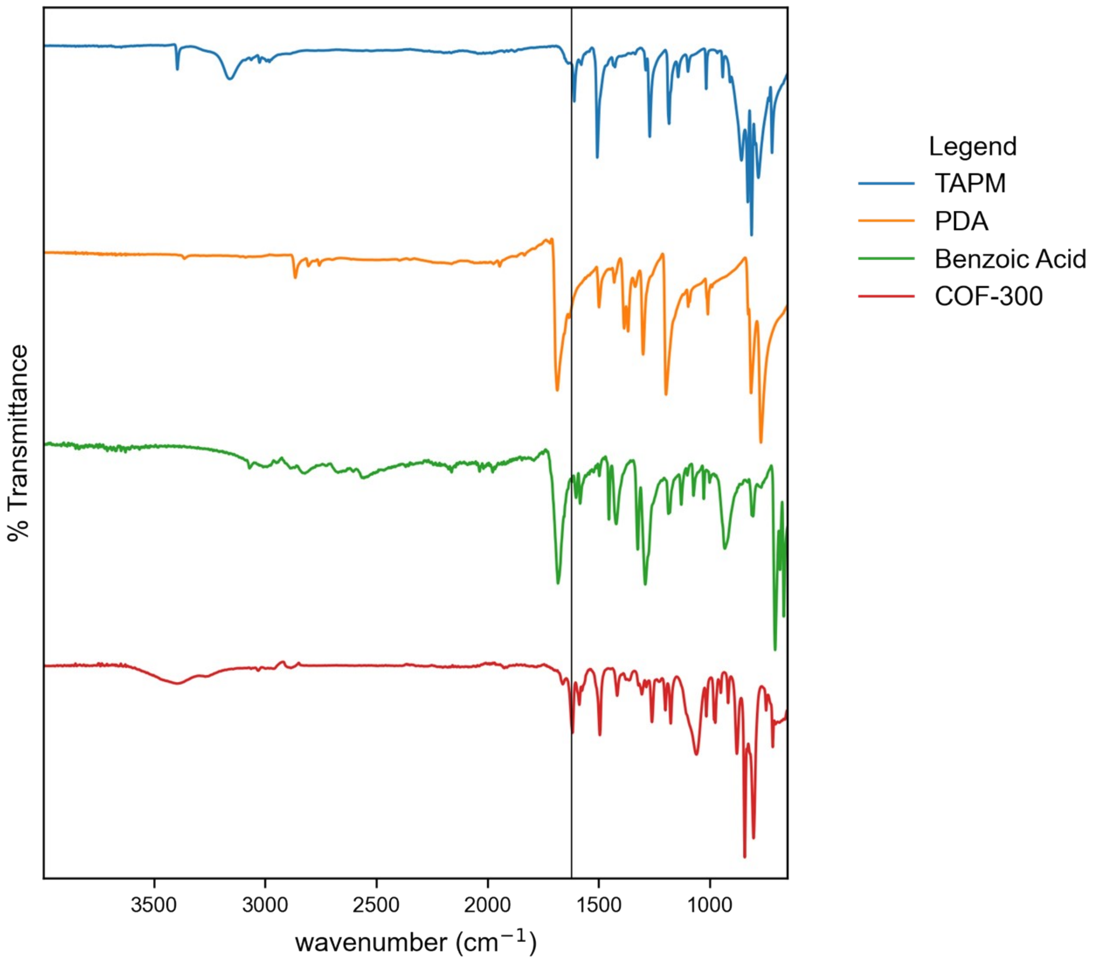

Figure S2. FTIR spectra for COF-300 monomers TAPM (blue), PDA (orange), benzoic acid catalyst (green), and isolated COF (red) shows the appearance of the C=N imine stretch at 1620.5 cm^{-1}. This signal is slightly overlapping with an aromatic ring-bending mode from TAPM which appears at 1612 cm^{-1}.[4]

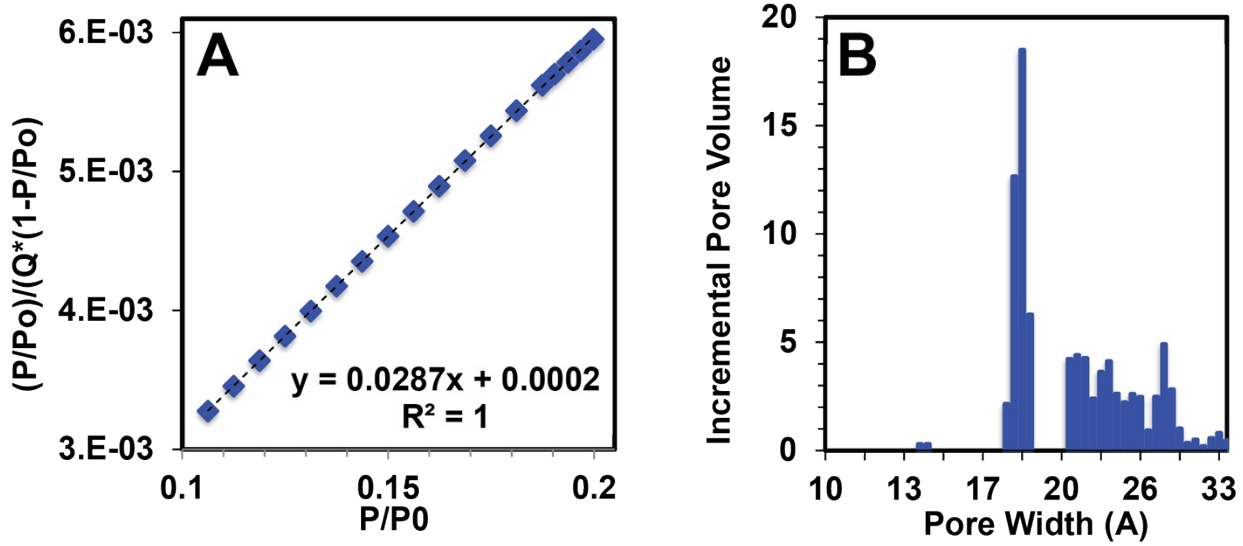

Figure S3. (A) BET plot and (B) pore size distribution of colloidally synthesized COF-300 with benzoic acid (**X**=H) as the imine condensation catalyst.

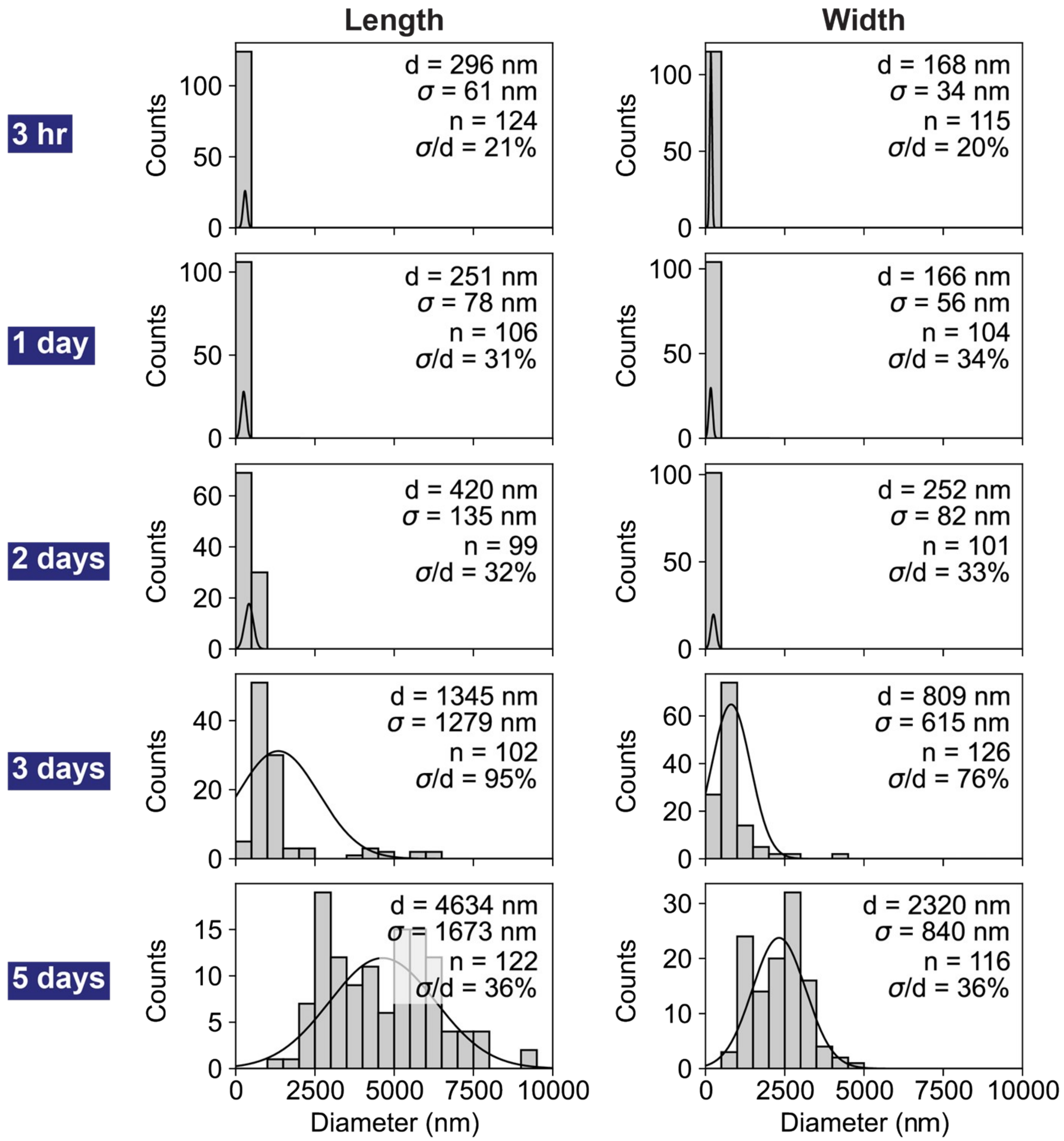

Figure S4. Histograms representing the dimensions of COF-300 colloids at different time points. Samples were synthesized in the presence of benzoic acid at 90 °C. Sizing was done in ImageJ.

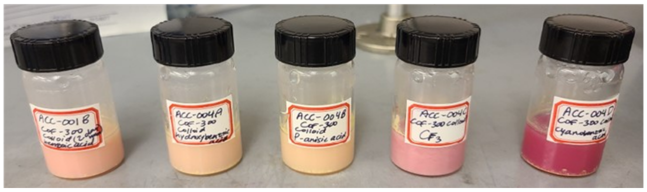

Figure S5. Appearance of COF-300 colloids after synthesis.

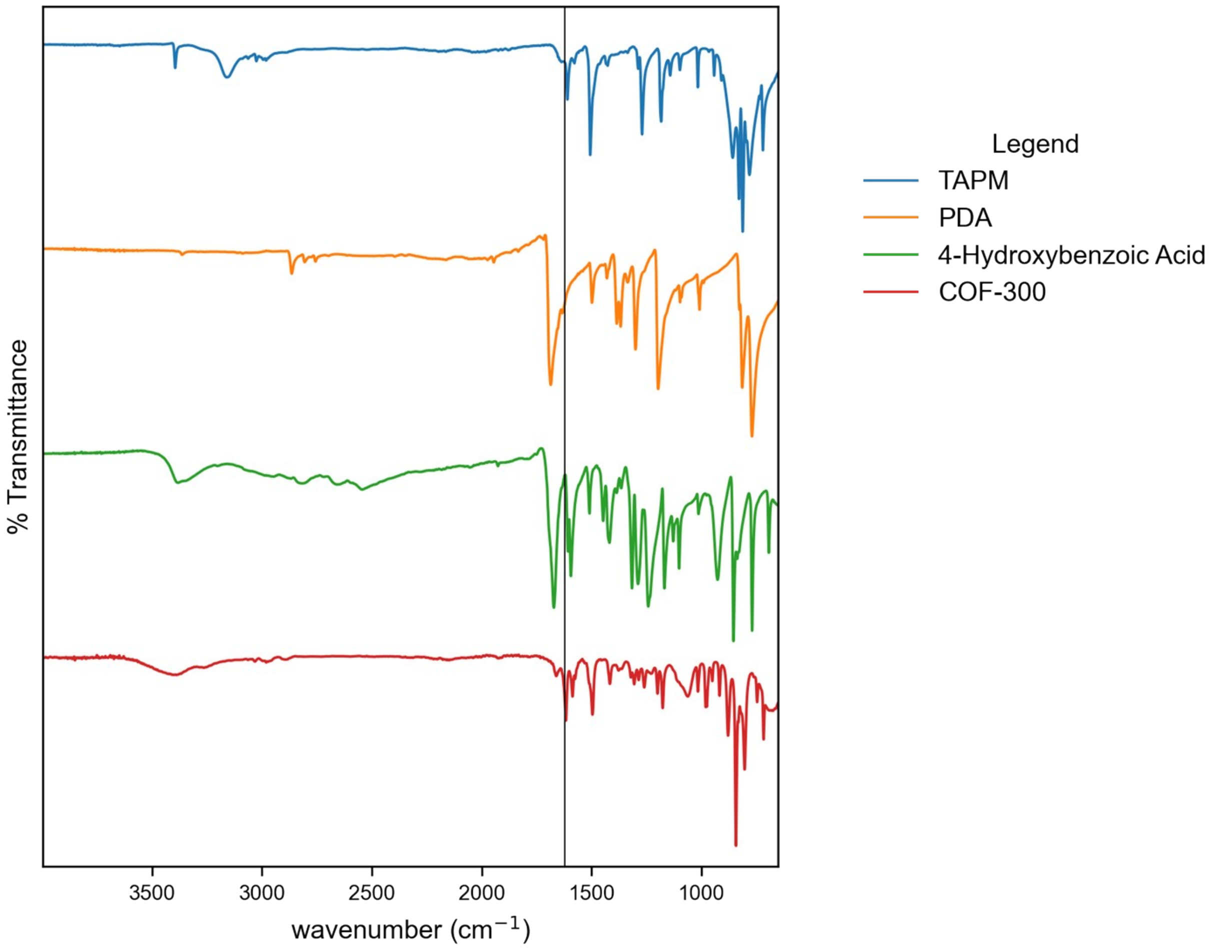

Figure S6. FTIR spectra for COF-300 monomers TAPM (blue), PDA (orange), 4-hydroxybenzoic acid catalyst (green), and isolated COF (red) shows the appearance of the C=N imine stretch at 1620.5 cm^{-1}. This signal is slightly overlapping with an aromatic ring-bending mode from TAPM which appears at 1612 cm^{-1}.[4]

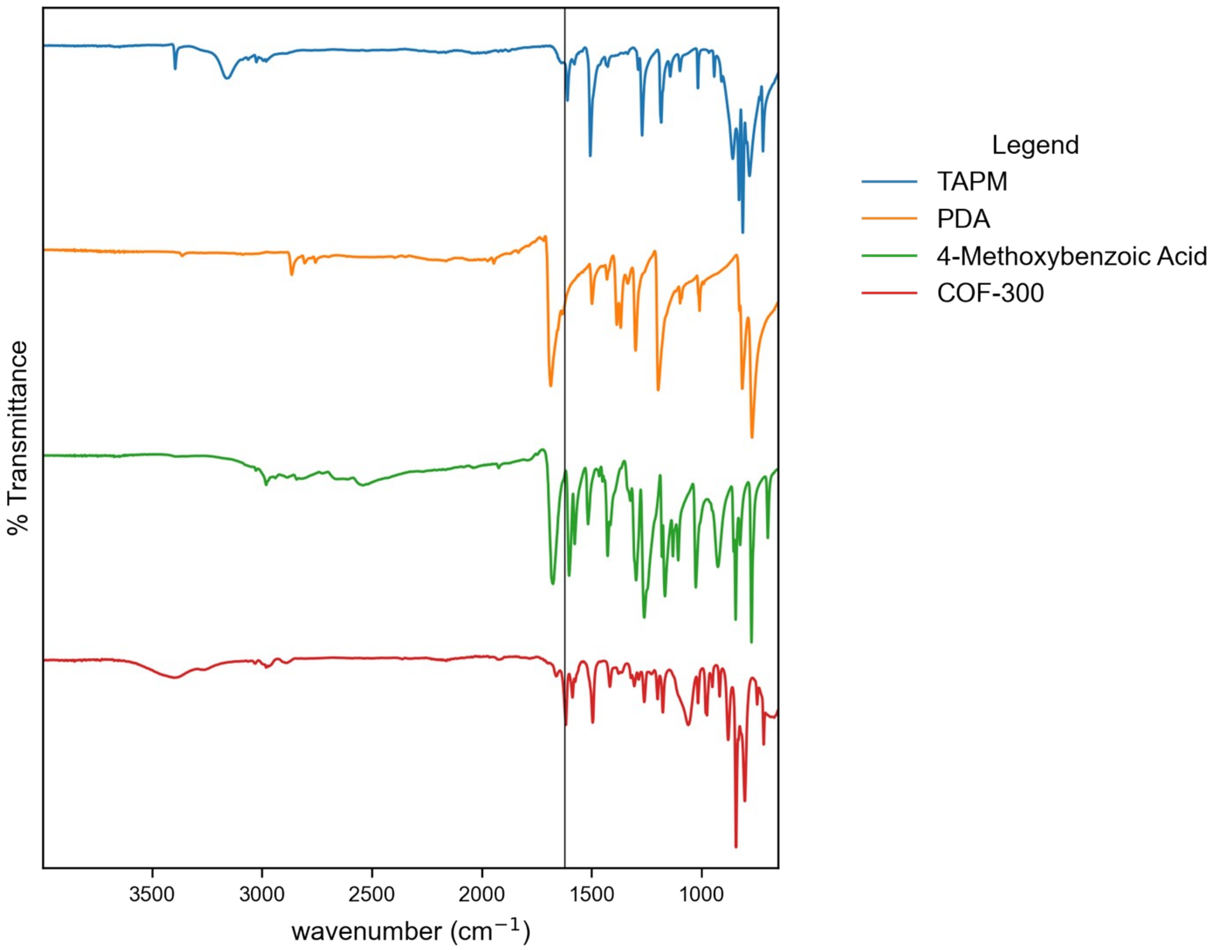

Figure S7. FTIR spectra for COF-300 monomers TAPM (blue), PDA (orange), 4-methoxybenzoic acid catalyst (green), and isolated COF (red) shows the appearance of the C=N imine stretch at 1620.5 cm^{-1}. This signal is slightly overlapping with an aromatic ring-bending mode from TAPM which appears at 1612 cm^{-1}.[4]

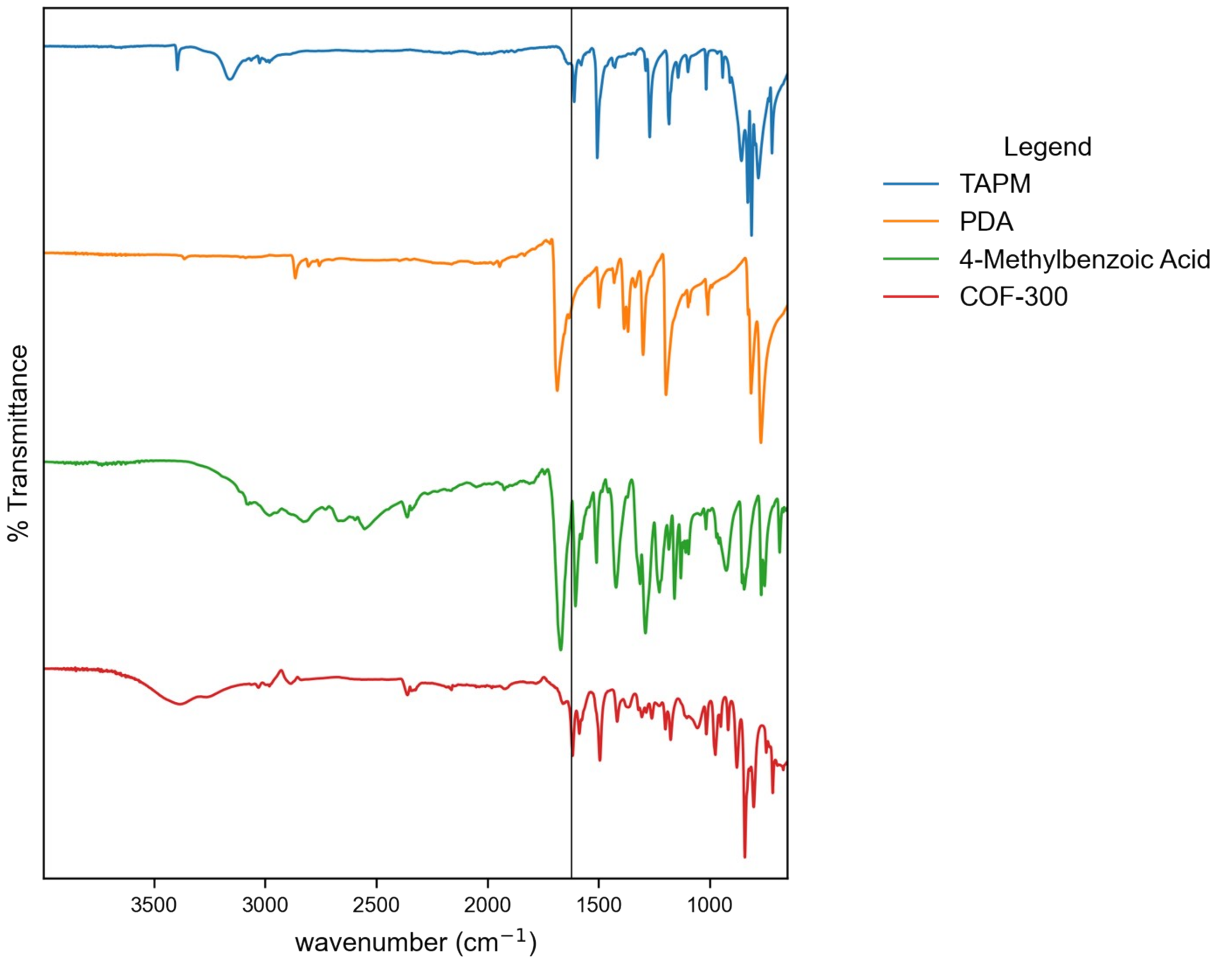

Figure S8. FTIR spectra for COF-300 monomers TAPM (blue), PDA (orange), 4-methylbenzoic acid catalyst (green), and isolated COF (red) shows the appearance of the C=N imine stretch at 1620.5 cm^{-1}. This signal is slightly overlapping with an aromatic ring-bending mode from TAPM which appears at 1612 cm^{-1}.[4]

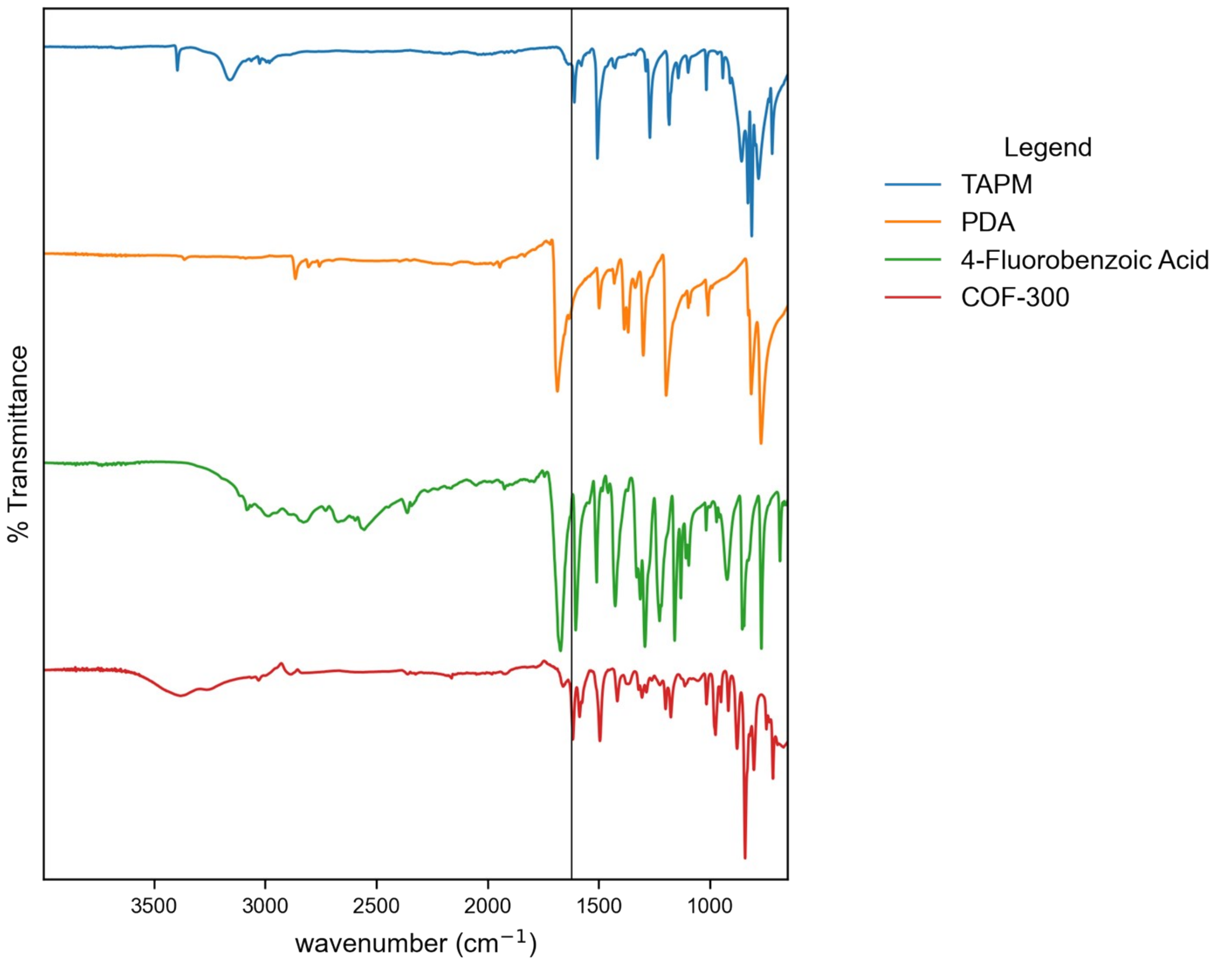

Figure S9. FTIR spectra for COF-300 monomers TAPM (blue), PDA (orange), 4-fluorobenzoic acid catalyst (green), and isolated COF (red) shows the appearance of the C=N imine stretch at 1620.5 cm^{-1}. This signal is slightly overlapping with an aromatic ring-bending mode from TAPM which appears at 1612 cm^{-1}.[4]

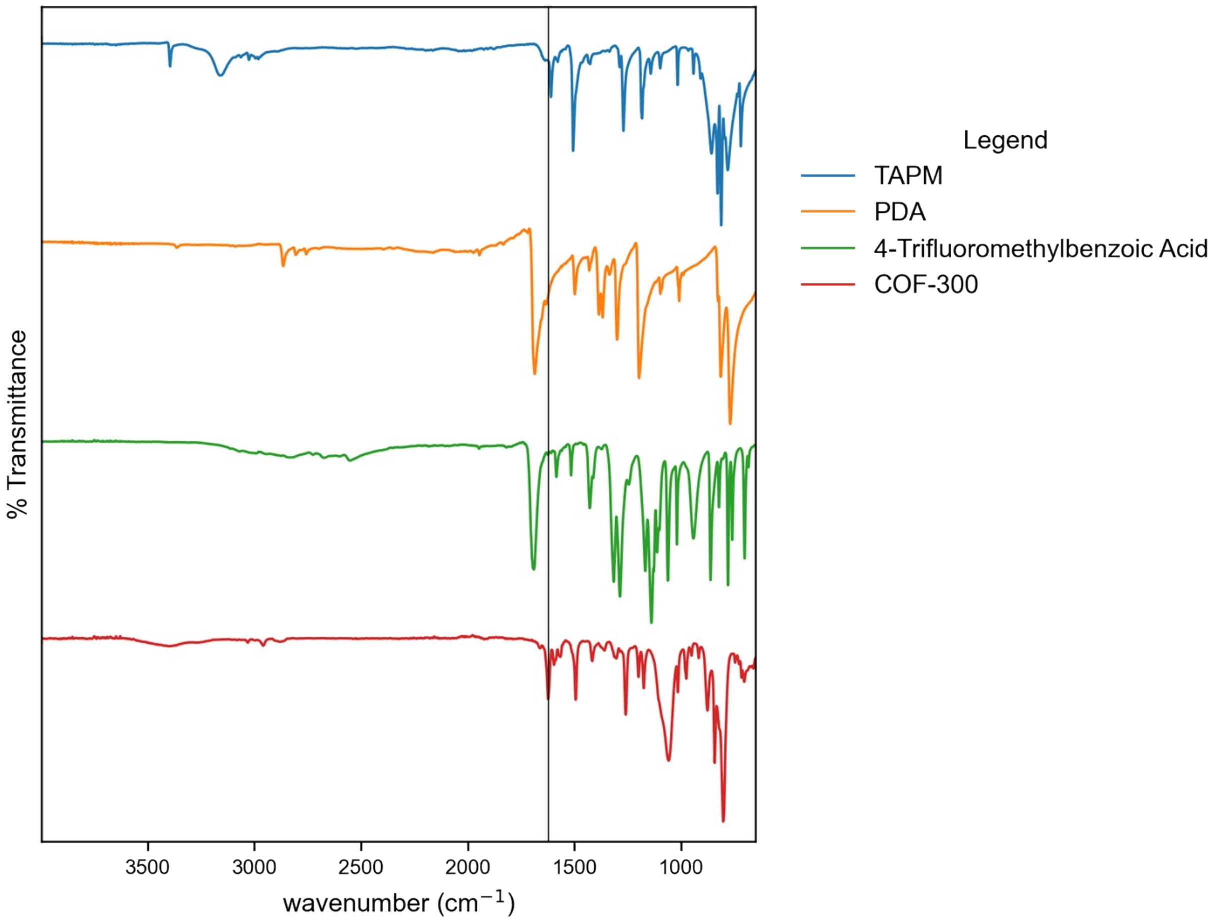

Figure S10. FTIR spectra for COF-300 monomers TAPM (blue), PDA (orange), 4-trifluoromethylbenzoic acid catalyst (green), and isolated COF (red) shows the appearance of the C=N imine stretch at 1620.5 cm^{-1}. This signal is slightly overlapping with an aromatic ring-bending mode from TAPM which appears at 1612 cm^{-1}.[4]

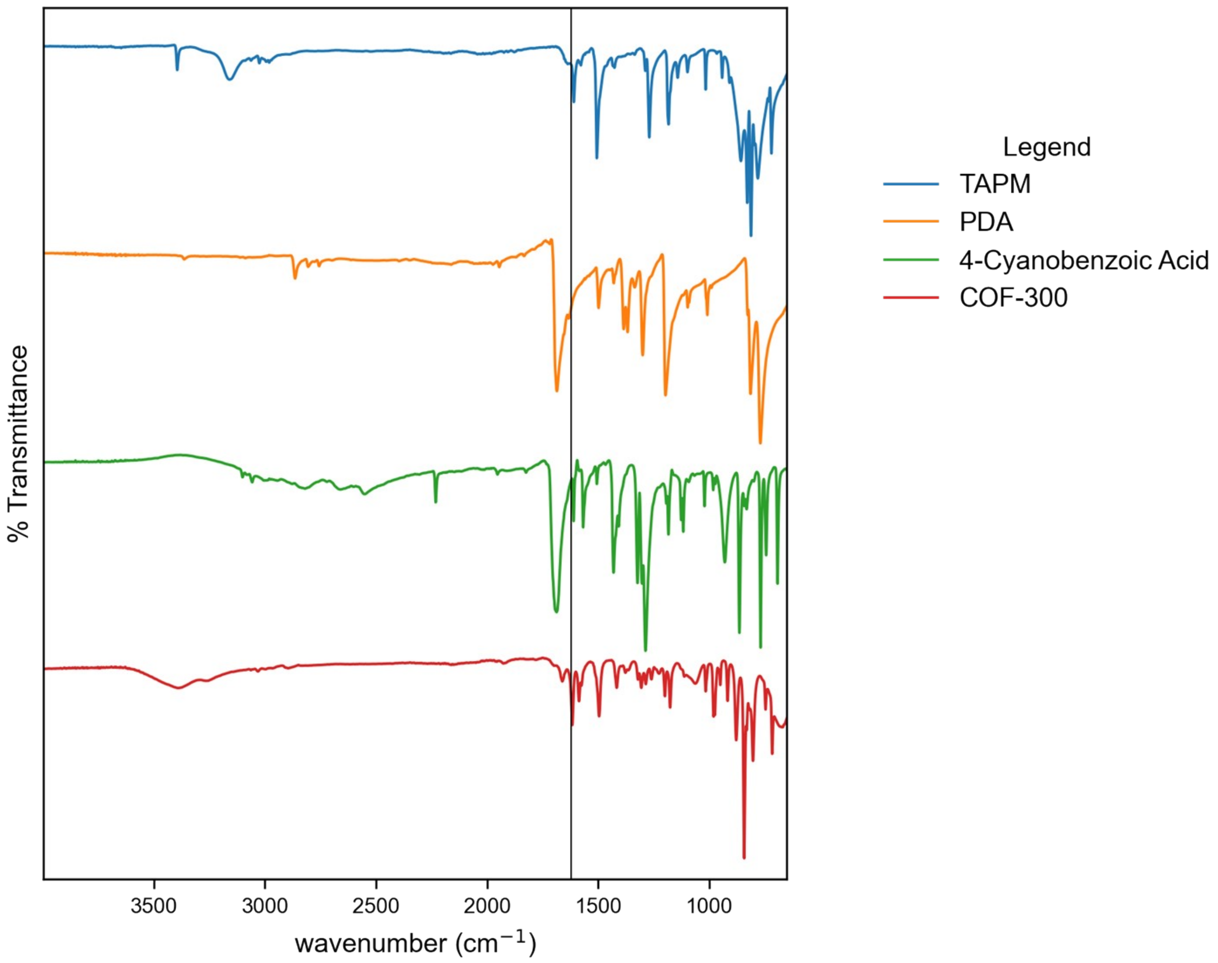

Figure S11. FTIR spectra for COF-300 monomers TAPM (blue), PDA (orange), 4-cyanobenzoic acid catalyst (green), and isolated COF (red) shows the appearance of the C=N imine stretch at 1620.5 cm^{-1}. This signal is slightly overlapping with an aromatic ring-bending mode from TAPM which appears at 1612 cm^{-1}.[4]

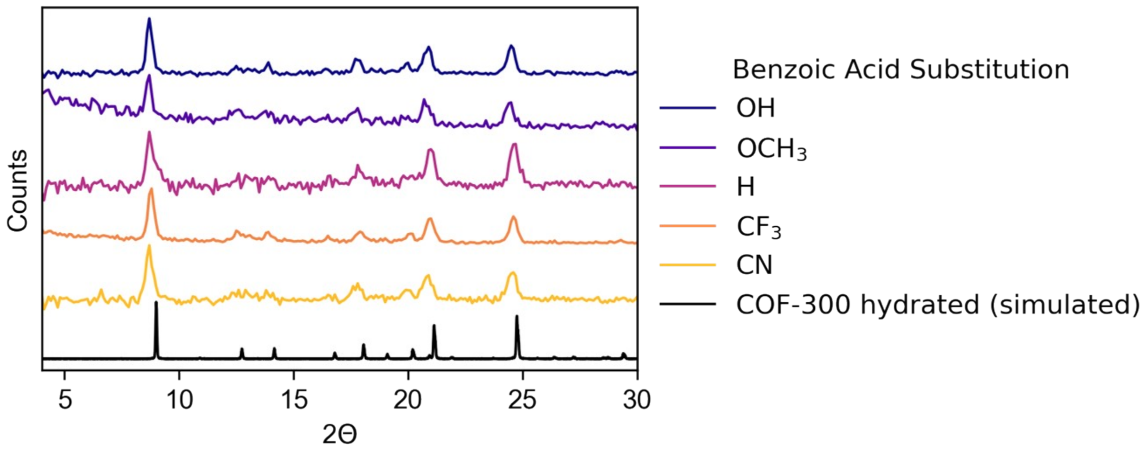

Figure S12. X-ray diffraction patterns for COF-300 samples synthesized with different substituted benzoic acids (**X** = OH, OCH_3, H, CF_3, CN) as compared to the simulated pattern.[5] Measured on a Siemens D5000 X-ray Diffractometer.

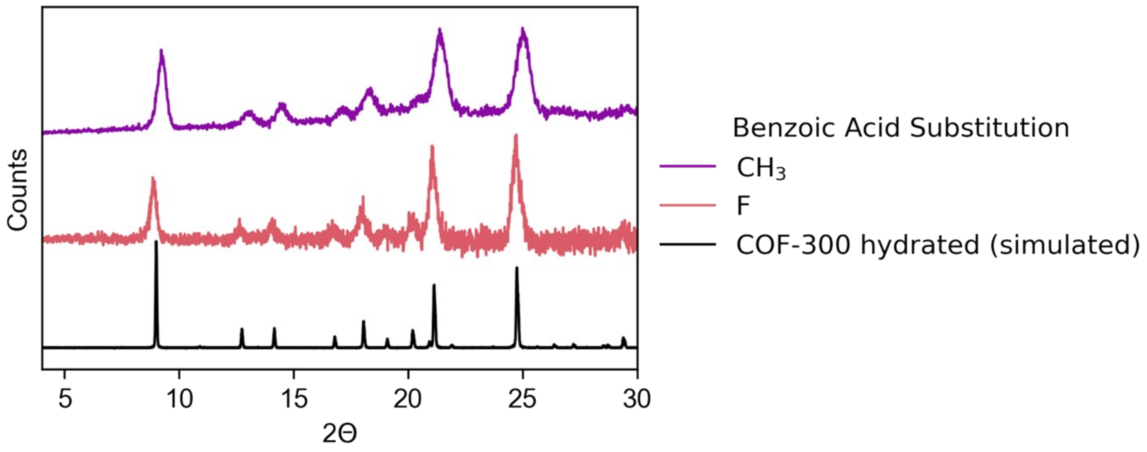

Figure S13. X-ray diffraction patterns for COF-300 samples synthesized with different substituted benzoic acids (**X** = CH_3, F) as compared to the simulated pattern.[5] Measured on a Rigaku MiniFlex-600.

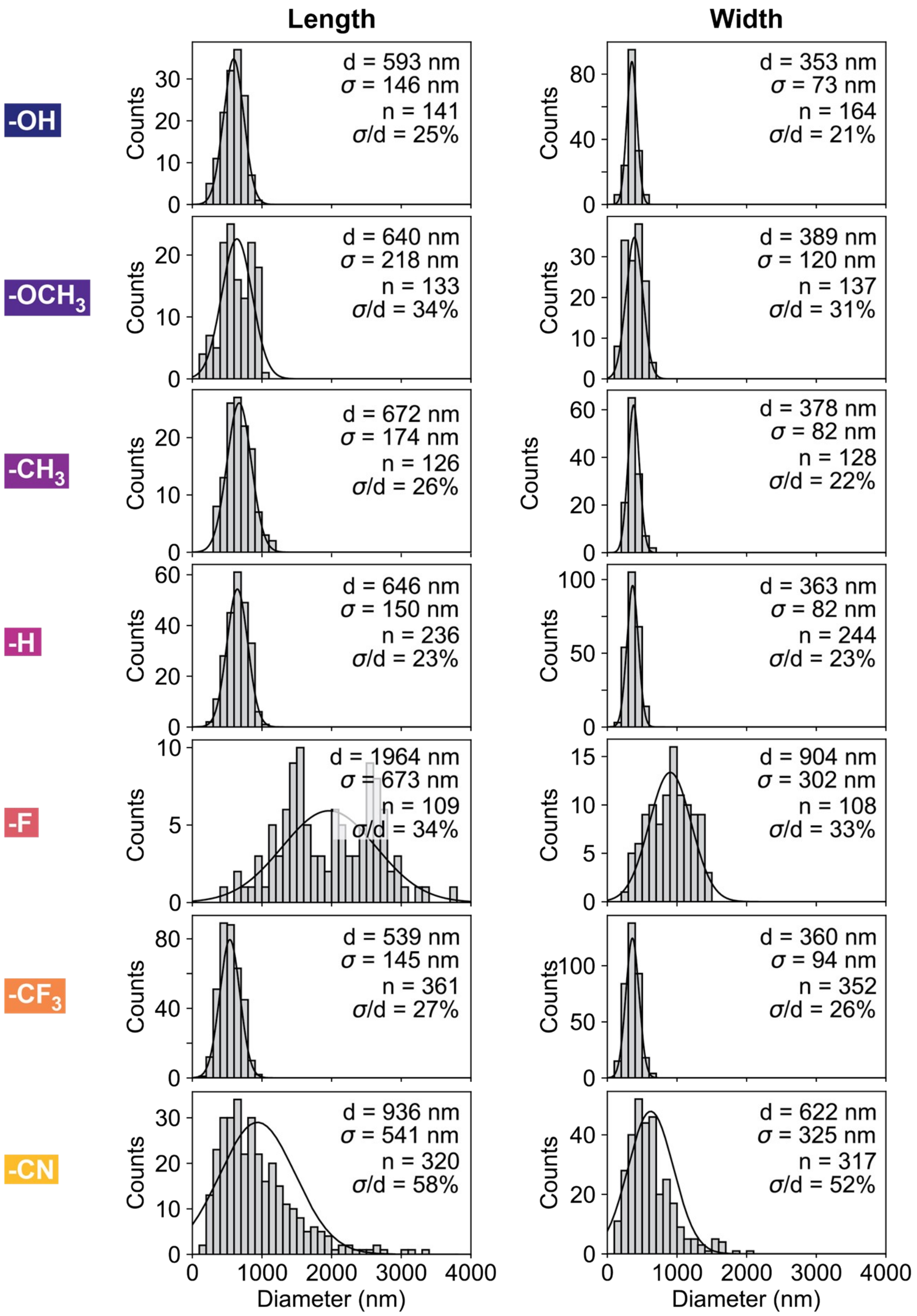

Figure S14. Histograms representing the dimensions of COF-300 colloids (synthesized in the presence of para-substituted benzoic acids) at 90 °C. Sizing was done in ImageJ.

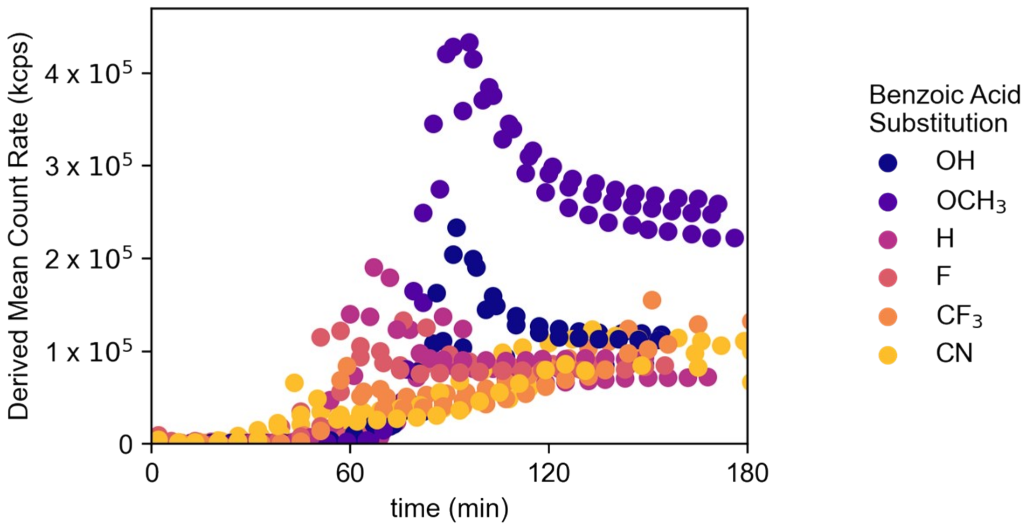

Figure S15. *In situ* dynamic light scattering data for COF-300 colloids synthesized with substituted benzoic acids (R = OH, OCH_3, H, F, CF_3, CN). Data shown comes from three trials per substituted benzoic acid. Although reactions are typically run for 48 hours, *in situ* data was collected for the first 1-2.5 hours of the reaction primarily to monitor time to nucleation as measured by an increase in derived mean count rate.

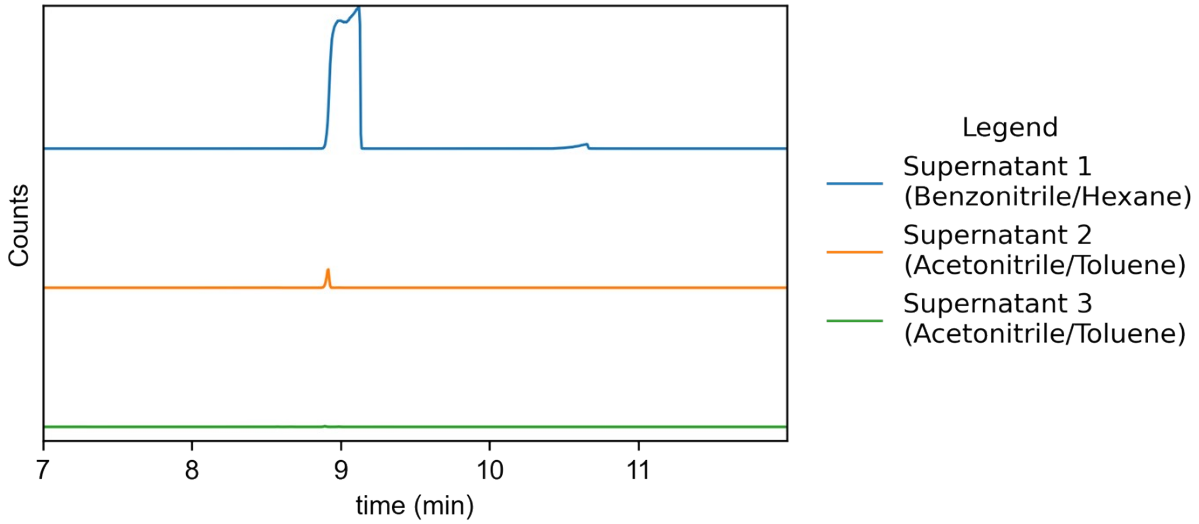

Figure S16. GC/MS of supernatants during COF-300 centrifugation purification show the initial presence of benzonitrile, aniline, and benzoic acid. After three rounds of purification, supernatant 3 shows only trace benzonitrile.

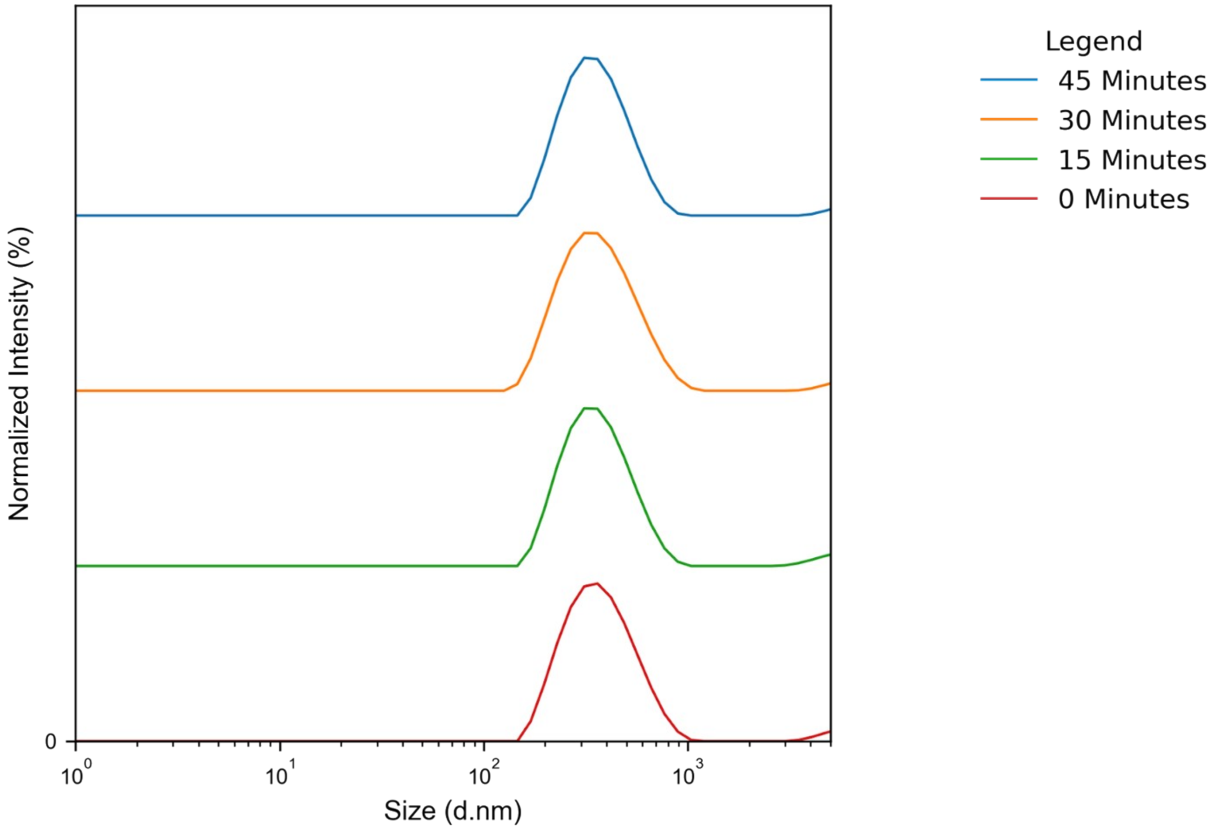

Figure S17. Dynamic light scattering data showing the normalized the size distribution by intensity of purified COF-300 colloid resuspended in 0.05 M benzoic acid in acetonitrile. The sample was measured at 15 minute intervals for 45 minutes.

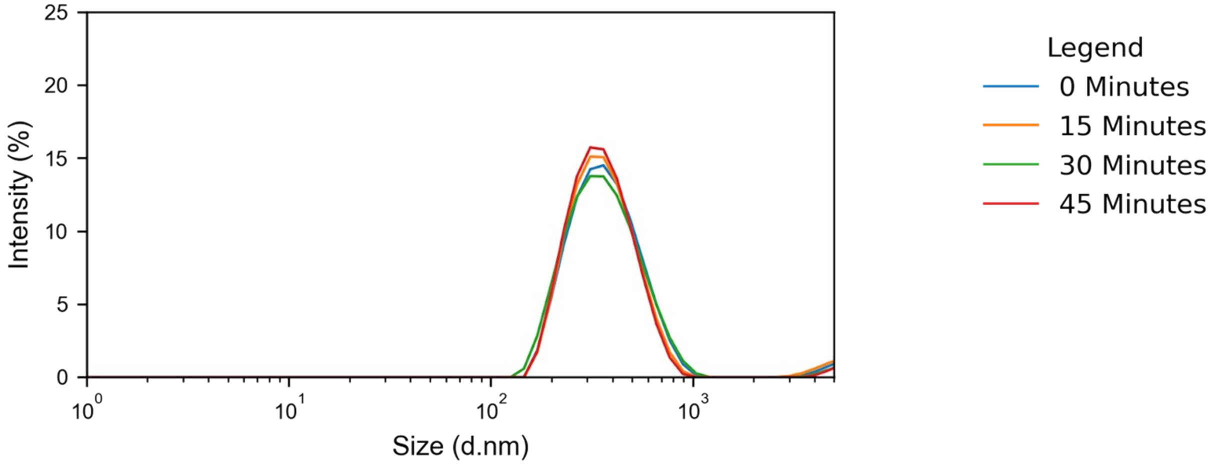

Figure S18. Dynamic light scattering data showing the size distribution by intensity of purified COF-300 colloid resuspended in 0.05 M benzoic acid in acetonitrile. The sample was measured at 15 minute intervals for 45 minutes.

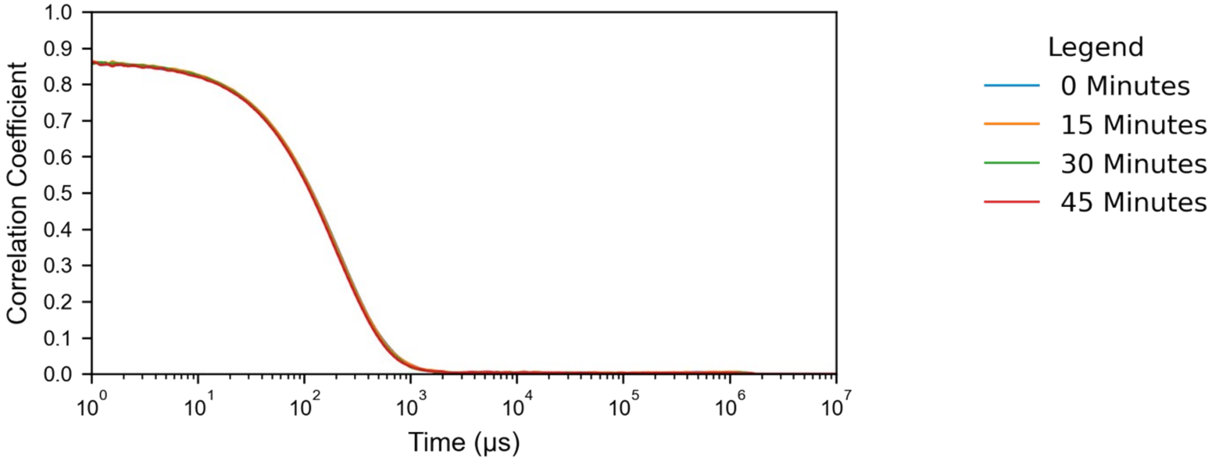

Figure S19. Dynamic light scattering data showing the correlogram of purified COF-300 colloid resuspended in 0.05 M benzoic acid in acetonitrile. The sample was measured at 15 minute intervals for 45 minutes.

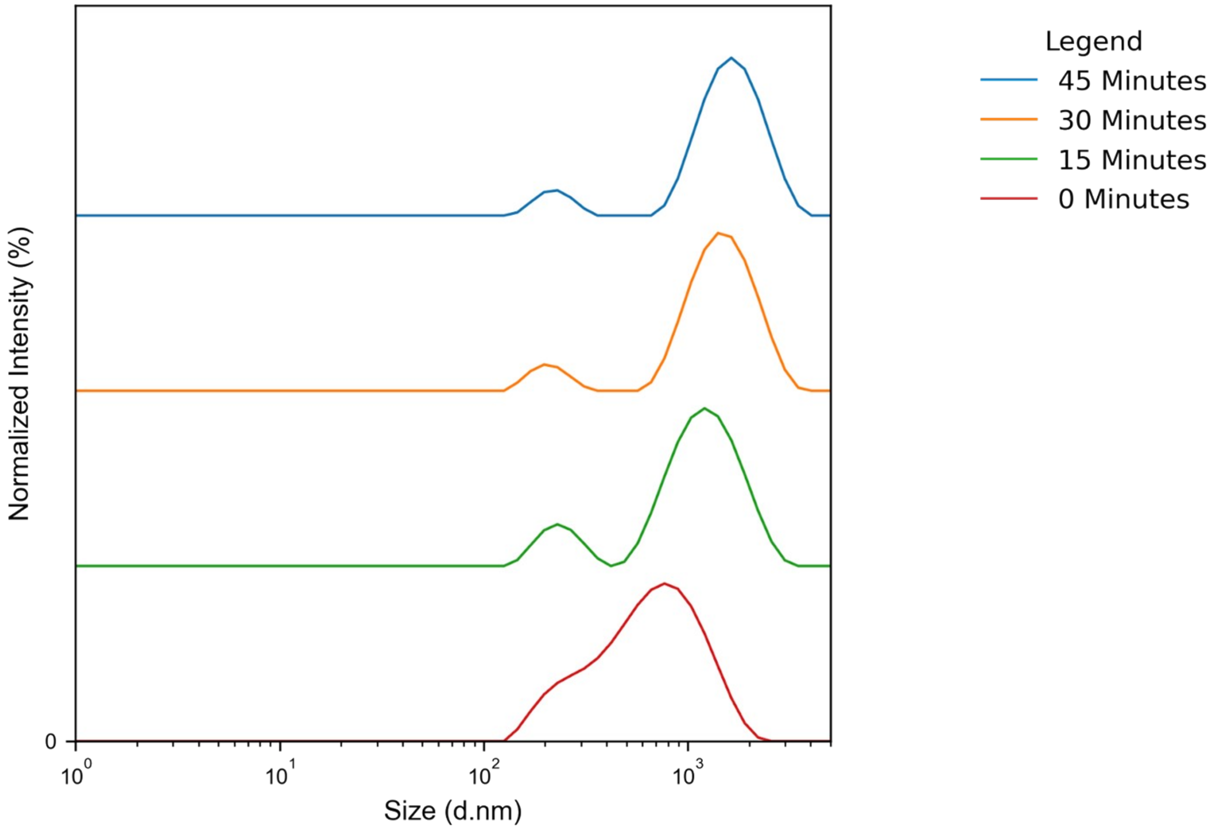

Figure S20. Dynamic light scattering data showing the normalized the size distribution by intensity of purified COF-300 colloid resuspended in 0.025 M benzoic acid in acetonitrile. The sample was measured at 15 minute intervals for 45 minutes.

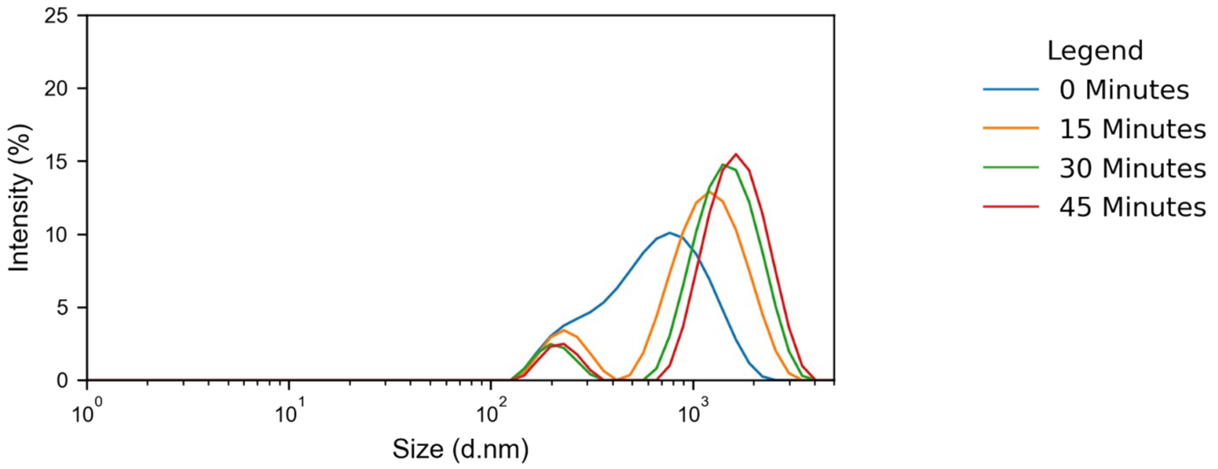

Figure S21. Dynamic light scattering data showing the size distribution by intensity of purified COF-300 colloid resuspended in 0.025 M benzoic acid in acetonitrile. The sample was measured at 15 minute intervals for 45 minutes.

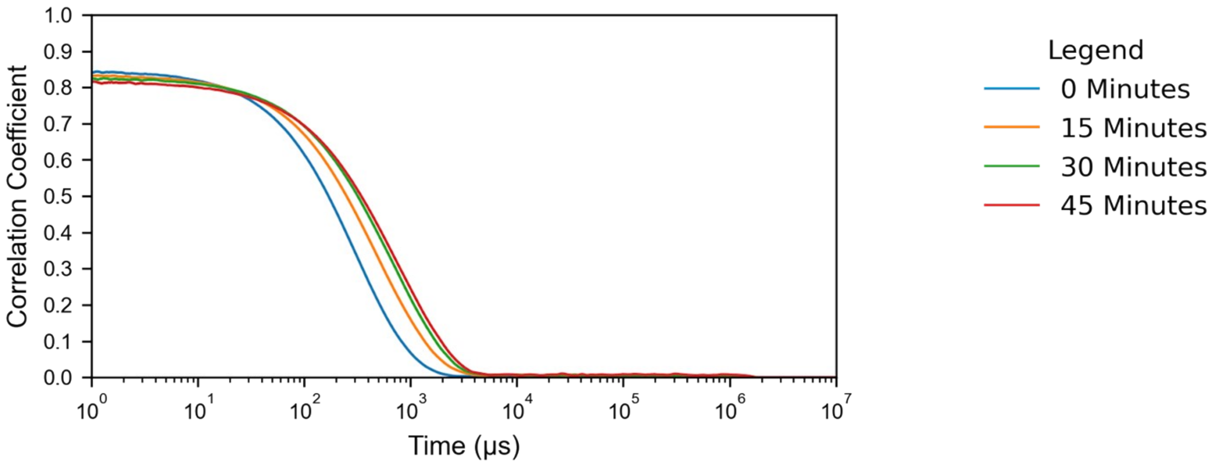

Figure S22. Dynamic light scattering data showing the correlogram of purified COF-300 colloid resuspended in 0.025 M benzoic acid in acetonitrile. The sample was measured at 15 minute intervals for 45 minutes.

Table S3. Benzoic acid derivative solubility in benzonitrile.

	4-hydroxy benzoic acid	4-methoxy benzoic acid	4-methyl benzoic acid	Benzoic acid	4-fluoro benzoic acid	4-(trifluoromethyl) benzoic acid	4-cyano benzoic acid
Max concentration in benzonitrile (M)	0.0818 M	0.0469 M	0.108 M	0.426 M	0.113 M	0.0402 M	0.0647 M

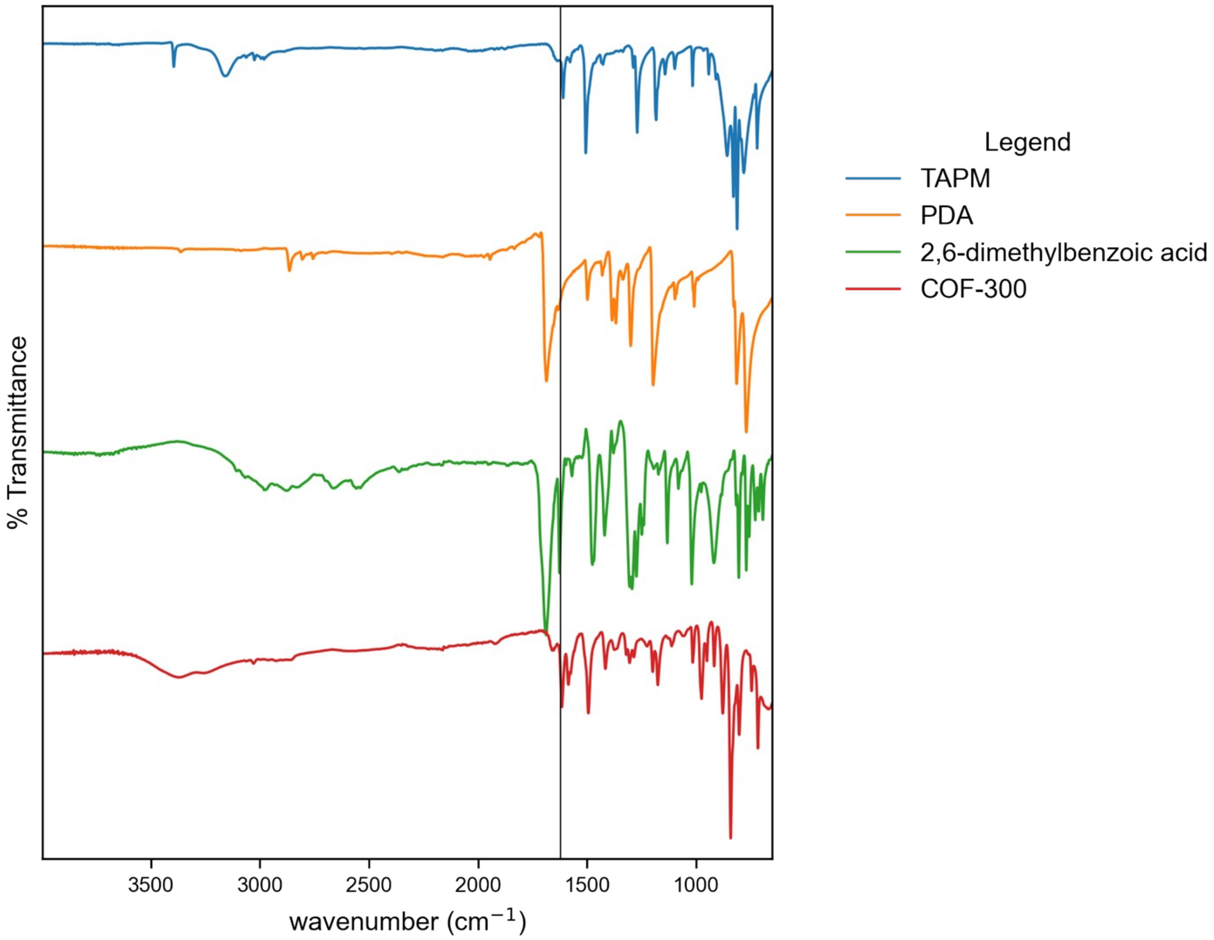

Figure S23. FTIR spectra for COF-300 monomers TAPM (blue), PDA (orange), 2,6-dimethylbenzoic acid catalyst (green), and isolated COF (red) shows the appearance of the C=N imine stretch at 1620.5 cm^{-1}. This signal is slightly overlapping with other signals including an aromatic ring-bending mode from TAPM which appears at 1612 cm^{-1}.[4]

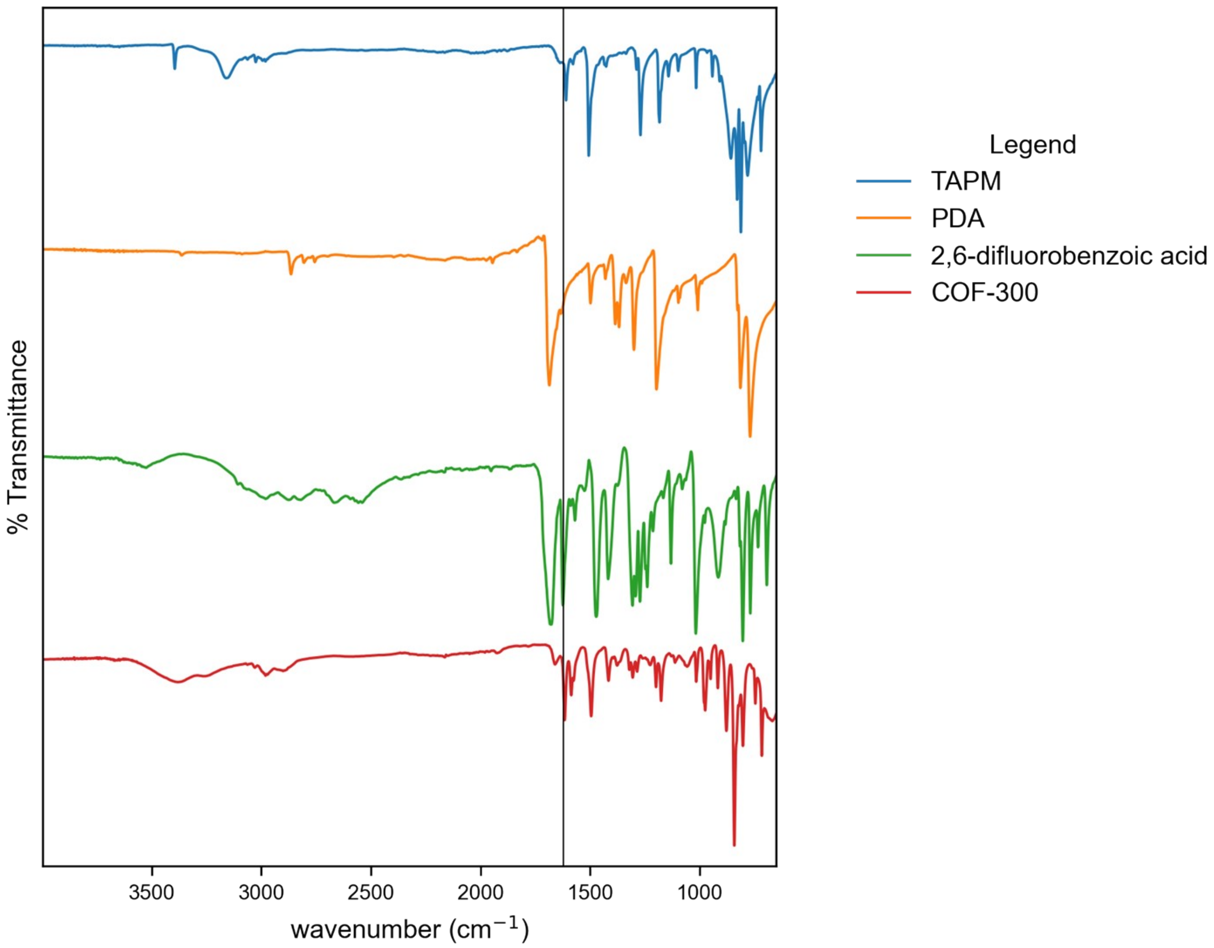

Figure S24. FTIR spectra for COF-300 monomers TAPM (blue), PDA (orange), 2,6-difluorobenzoic acid catalyst (green), and isolated COF (red) shows the appearance of the C=N imine stretch at 1620.5 cm^{-1}. This signal is slightly overlapping with other signals including an aromatic ring-bending mode from TAPM which appears at 1612 cm^{-1}.[4]

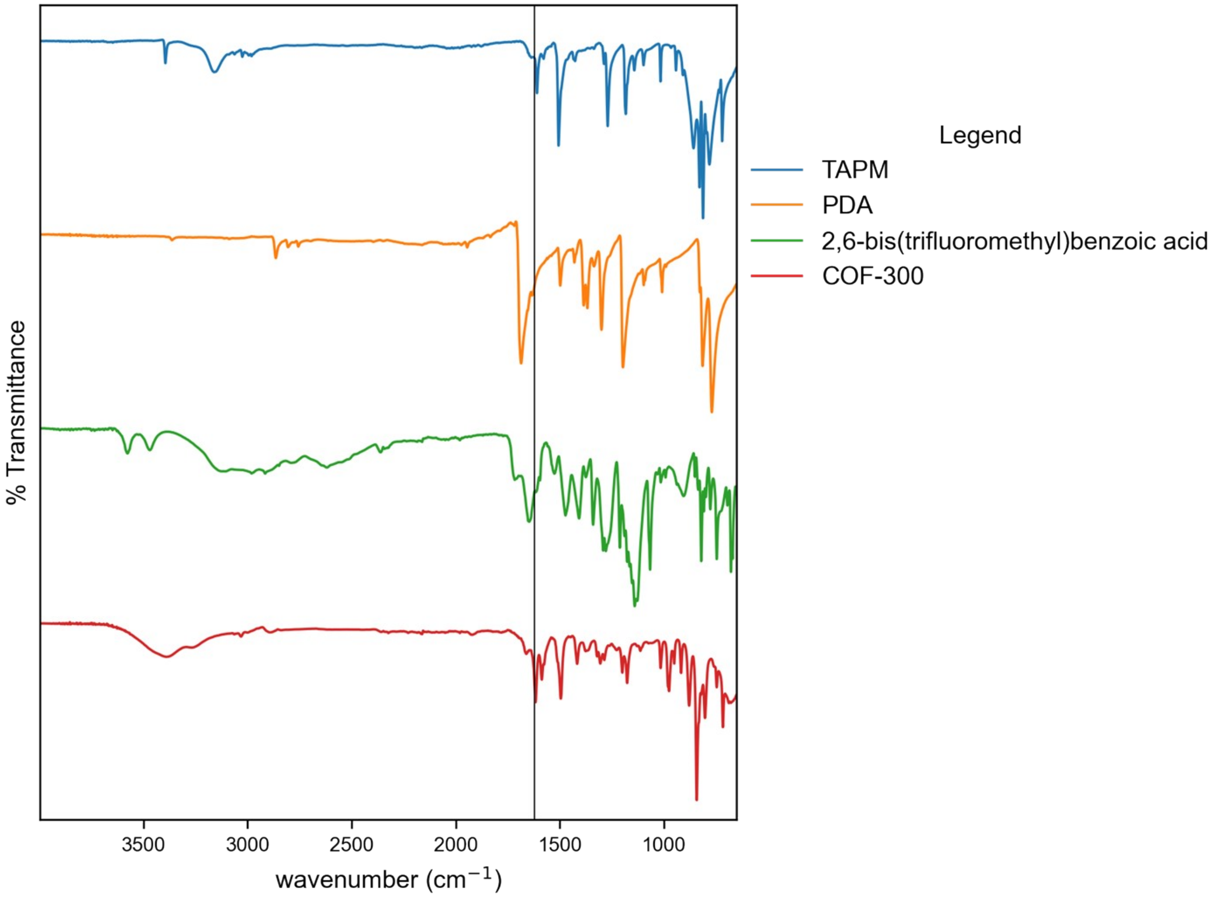

Figure S25. FTIR spectra for COF-300 monomers TAPM (blue), PDA (orange), 2,6-bis(trifluoromethyl)benzoic acid catalyst (green), and isolated COF (red) shows the appearance of the C=N imine stretch at 1620.5 cm^{-1}. This signal is slightly overlapping with other signals including an aromatic ring-bending mode from TAPM which appears at 1612 cm^{-1}.[4]

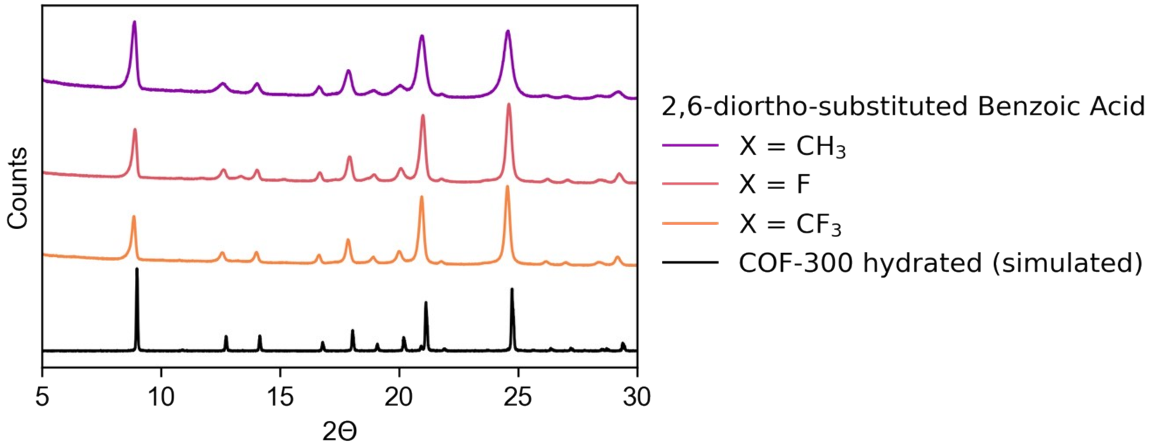

Figure S26. X-ray diffraction patterns for COF-300 samples synthesized with different diortho-substituted benzoic acids (**X** = CH_3, F, CF_3) as compared to the simulated pattern.[5] Measured on a Panalytical Empyrean Powder Diffractometer.

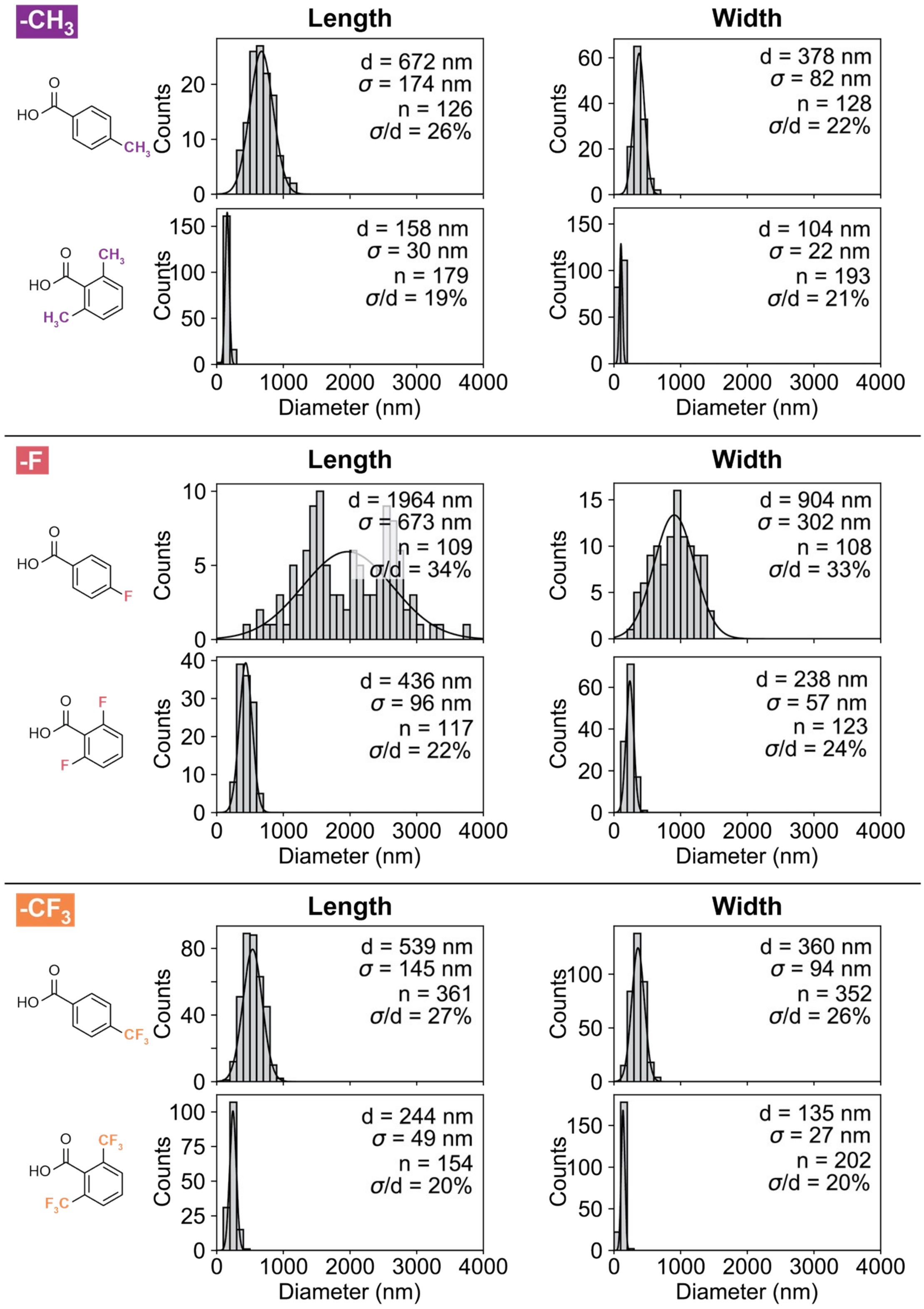

Figure S27. Histograms representing the dimensions of COF-300 colloids (synthesized in the presence of para-substituted and diortho-substituted benzoic acids (**X** = CH_3, F, CF_3)) at 90 °C. SEM images shown in Figure 7. Sizing was done in ImageJ.

E. Pair Distribution Function Analysis

The real space pair distribution function $G(r)$ measures the deviation from the average number density p_0 at a pair distance r and represents a histogram of interatomic distances in a system.

$$G(r) = 4\pi r(\rho(r) - p_0)$$

$G(r)$ is obtained as the sine fourier transform of the normalized scattering intensity $S(Q)$

$$G(r) = \frac{2}{\pi}\int_{Q_{min}}^{Q_{max}} Q(S(Q) - 1)\sin(Qr)dQ$$

From a periodic model the PDF is calculated as

$$G(r)_{calc} = \frac{1}{Nr}\sum_{i}\sum_{i\neq j}\left[\frac{f_i f_j}{\langle f\rangle^2} e^{-\frac{(r - r_{ij})^2}{\sigma_{ij}}}\right] - 4\pi r p_0$$

With the peak width σ_{ij} depending on the atomic displacement parameters of the two atoms i,j as well as correlated motion sharpening and beamline resolution broadening factors.[3]

Following previous work on molecular crystals, our calculated model G_{calc} accounting for different intermolecular and intramolecular ADPs is[6-7]

$$G_{calc} = a * (G_{intra(1.1\,Å - 5.0Å)} + G_{inter(1.1\,Å - 50.0Å)} - G_{inter(1.1\,Å - 5.0Å)})$$

Where a is a common scale factor and $intra$ and $inter$ are separately refined ADPs along the indicated real space distance ranges. We can visualize this model as shown below in Figure SX.

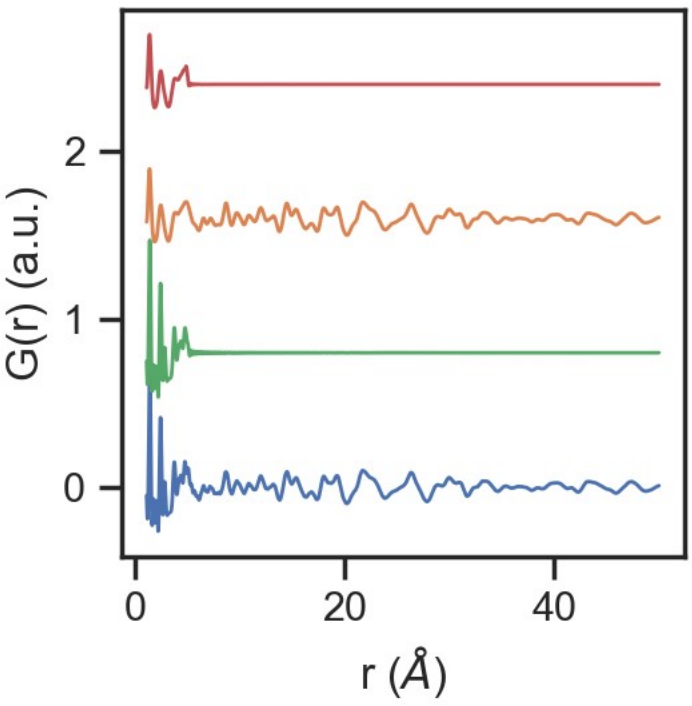

Figure S28. Visualization of phase components for COF PDF fitting where G_{total} is the blue trace, $G_{intra\ (1.1\ Å\ -\ 5.0\ Å\ )}$ is the green trace, $G_{inter\ (1.1\ Å\ -\ 5.0\ )}$ is the orange trace, and $G_{inter(1.1\ Å\ -\ 5.0Å\ )}$ is the red trace.

For each phase we use the hydrated COF-300 single crystal structure (Ma *et al.* CCDC number 1846136).[5] Refined parameters of fitting are summarized in tables below. H atom ADPs were not refined and due to their relatively small contribution to the total X-ray scattering and potential for overfitting.

Model and data for a conventional periodic one phase mode and model accounting for intermolecular and intramolecular ADPs is shown in Figure 2 of the manuscript. While the proxy model does not capture all features particularly at intermediate distance ranges around the cutoff for the two ADPs (5.0 Å) the improvement in fit quality is substantial (see tables below).

In total the fitting suggests that the crystalline 3D COFs synthesized here possess substantial disorder from their average structures as has been shown similarly for 2D COFs. While short length scales within the size of a subunit monomer are well ordered, substantial disorder exists in the 3D framework despite the COF showing good average crystallinity.

Box car fits over distance ranges from 1-22 angstroms highlight the sharp increase of the C ADP when going from intramonomer to intermonomer distance ranges as shown below. A sharp spike in the goodness of fit parameter is seen in the distance range where both intermonomer and intramonomer distances overlap and the two ADP proxy model is unsuitable.

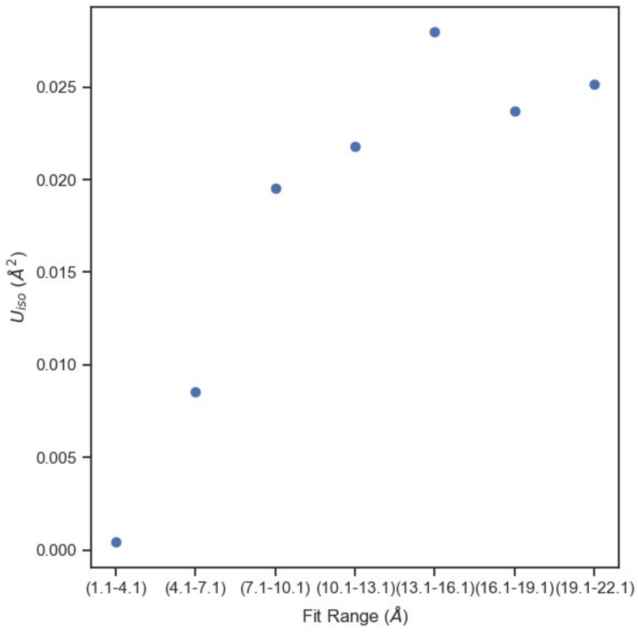

Figure S29. C,N U_{iso} values as a function of real space fit range for a one phase fit of COF

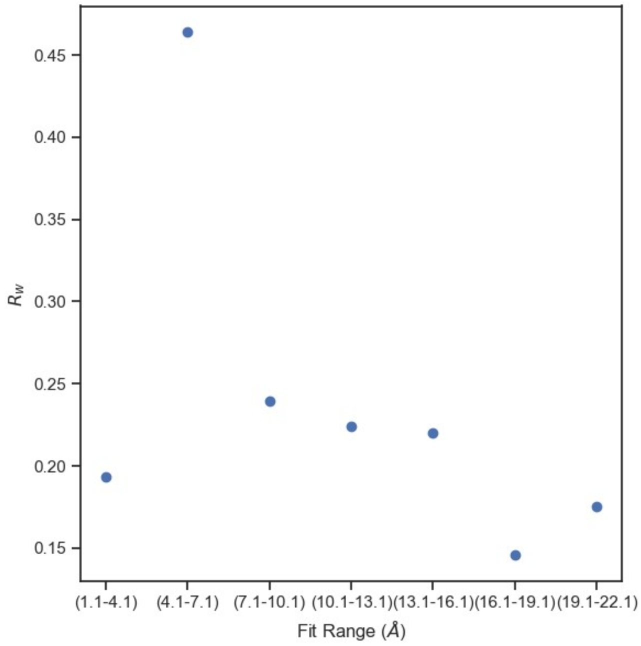

Figure S30. Corresponding R_w values for fits in figure above

One ADP	$Large$	$Small$
a (Å)	19.57	19.55
b (Å)	19.57	19.55
c (Å)	8.93	8.92
δ_2	1.16	1.16
Scale Factor	0.0699	0.0639
C,N U_{iso} ($Å^2$)	0.00530	0.00576
O U_{iso} ($Å^2$)	0.0970	0.134
rw	0.460	0.467

Table S4. Refined parameters of single phase fits to COF data for large (length = 936 nm) and small (length = 251 nm) COF-300 crystallites.

Two ADP	$Large$	$Small$
a (Å)	19.54	19.52
b (Å)	19.54	19.52
c (Å)	8.93	8.99
δ_2	1.16	1.16
Scale Factor	0.0757	0.0717
Intra C,N U_{iso} ($Å^2$)	0.000608	0.00107
Intra O U_{iso} ($Å^2$)	00.0302	0.0209
Inter C,N U_{iso} ($Å^2$)	0.0214	0.0276
Inter O U_{iso} ($Å^2$)	0.0969	0.1913
rw	0.256	0.257

Table S5. Refined parameters of multi-phase fits to COF data for large (length = 936 nm) and small (length = 251 nm) COF-300 crystallites.

E. ^{1}H NMR Spectra

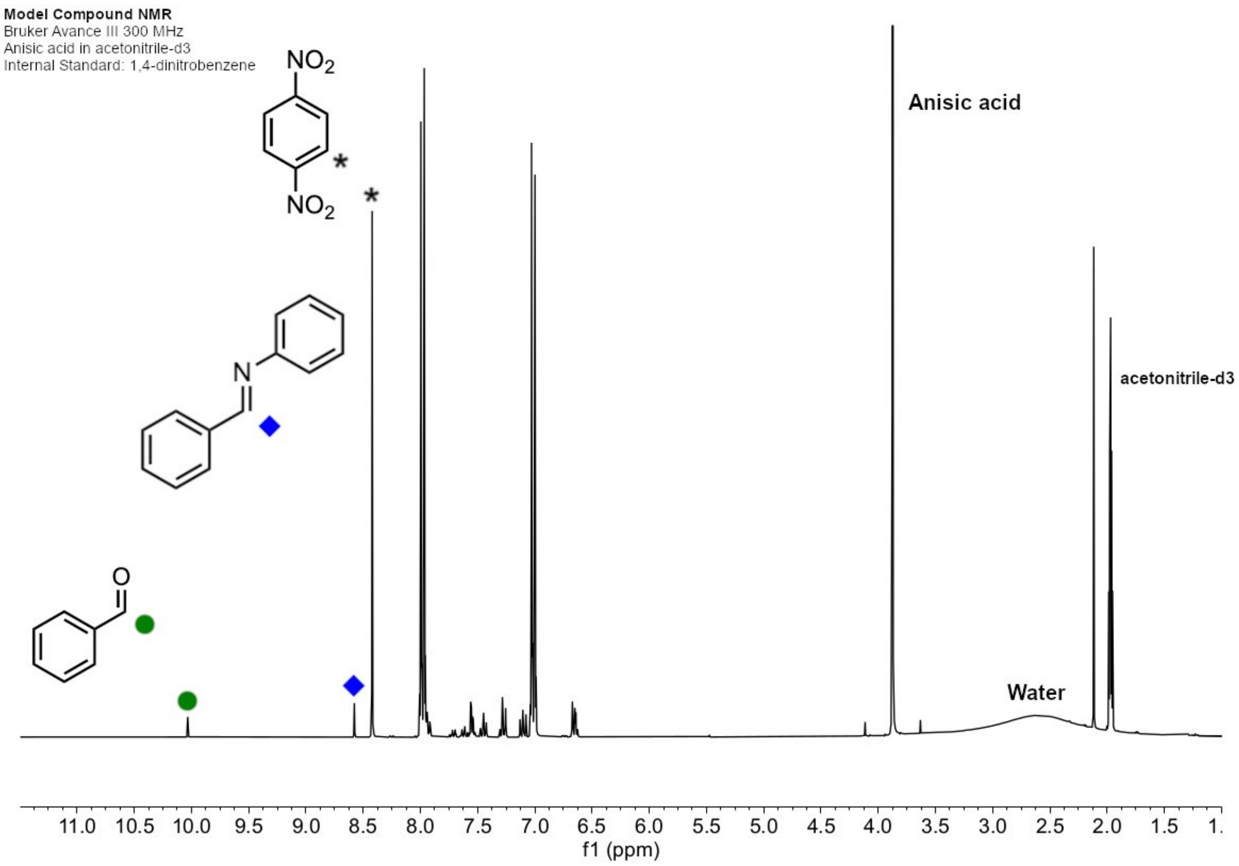

Figure S31. ^{1}H NMR spectrum of aniline + benzaldehyde + *p*-anisic acid (model compound reaction) in acetonitrile-d3.

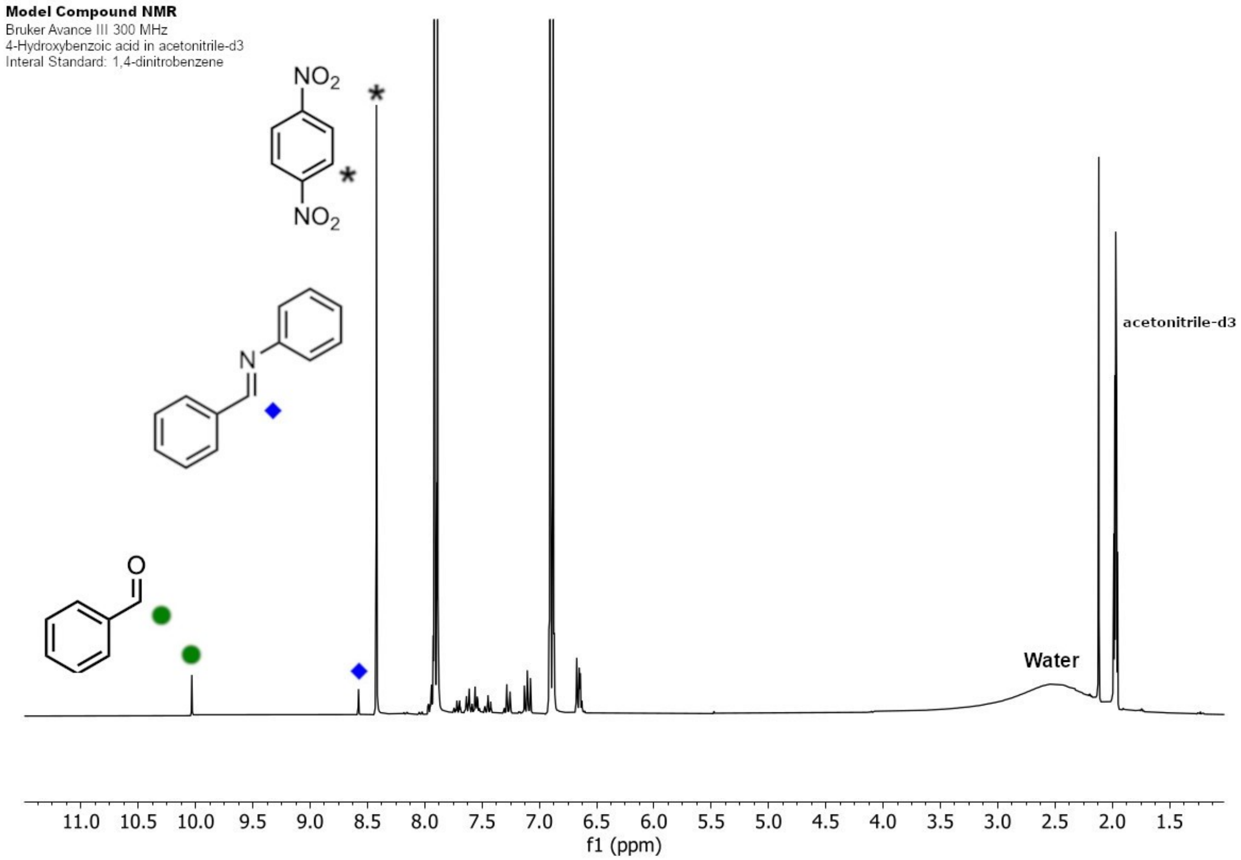

Figure S32. ^{1}H NMR spectrum of aniline + benzaldehyde + 4-hydroxybenzoic acid (model compound reaction) in acetonitrile-d3.

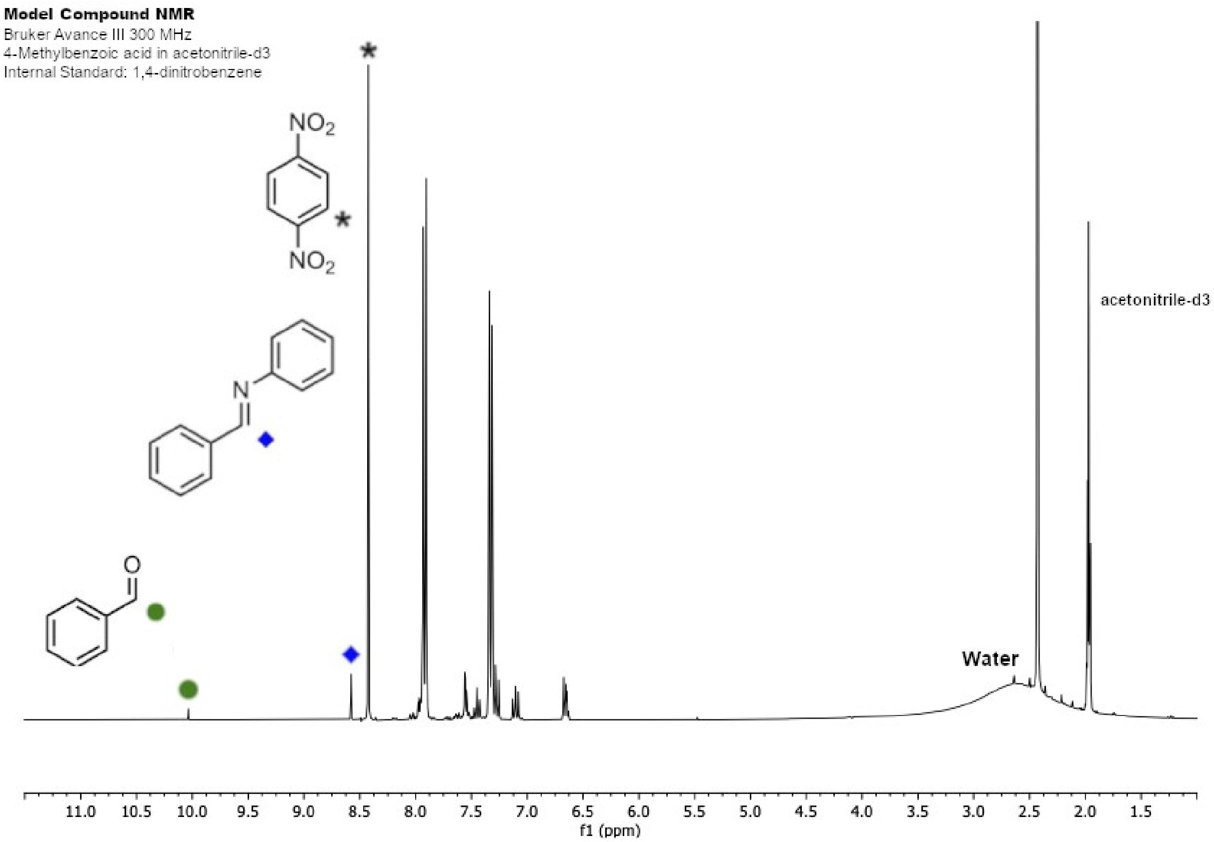

Figure S33. ^{1}H NMR spectrum of aniline + benzaldehyde + 4-methylbenzoic acid (model compound reaction) in acetonitrile-d3.

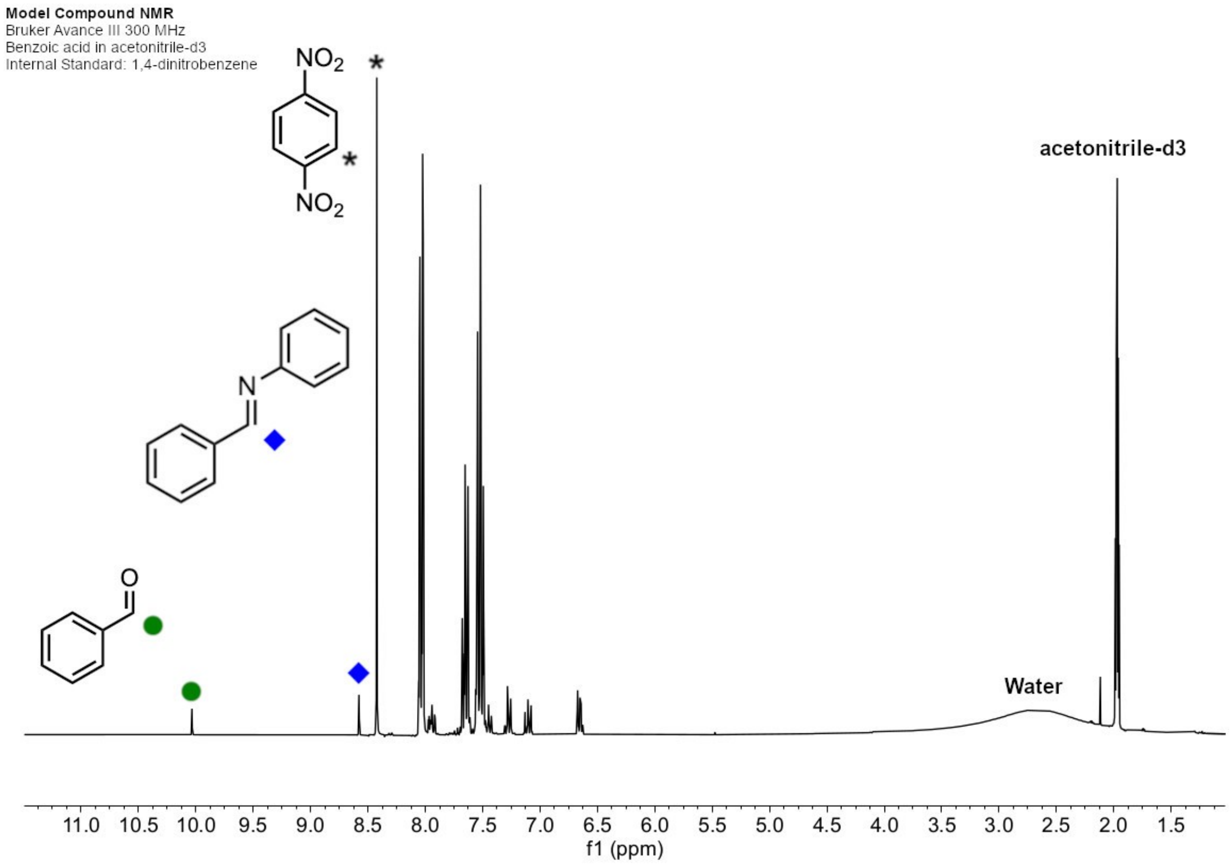

Figure S34. ^{1}H NMR spectrum of aniline + benzaldehyde + benzoic acid (model compound reaction) in acetonitrile-d3.

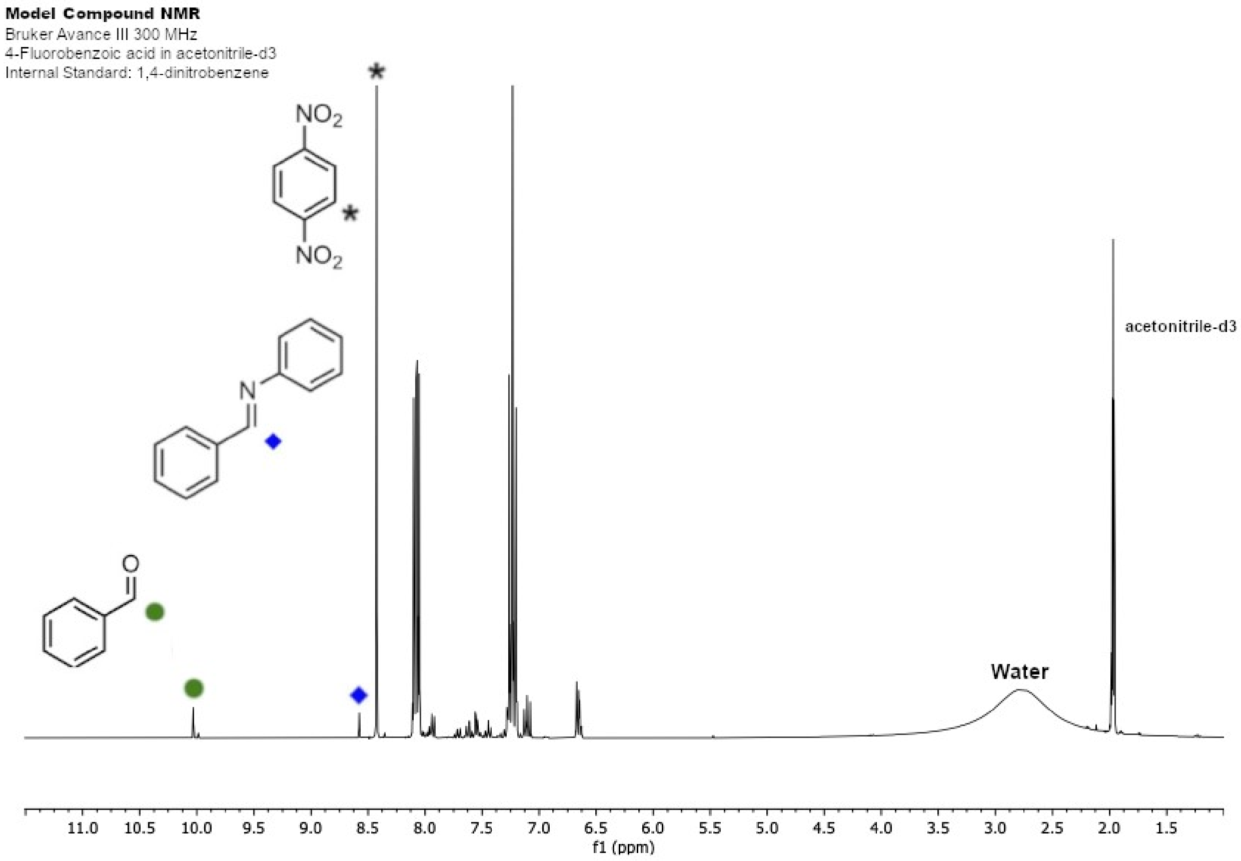

Figure S35. ^{1}H NMR spectrum of aniline + benzaldehyde + 4-fluorobenzoic acid (model compound reaction) in acetonitrile-d3.

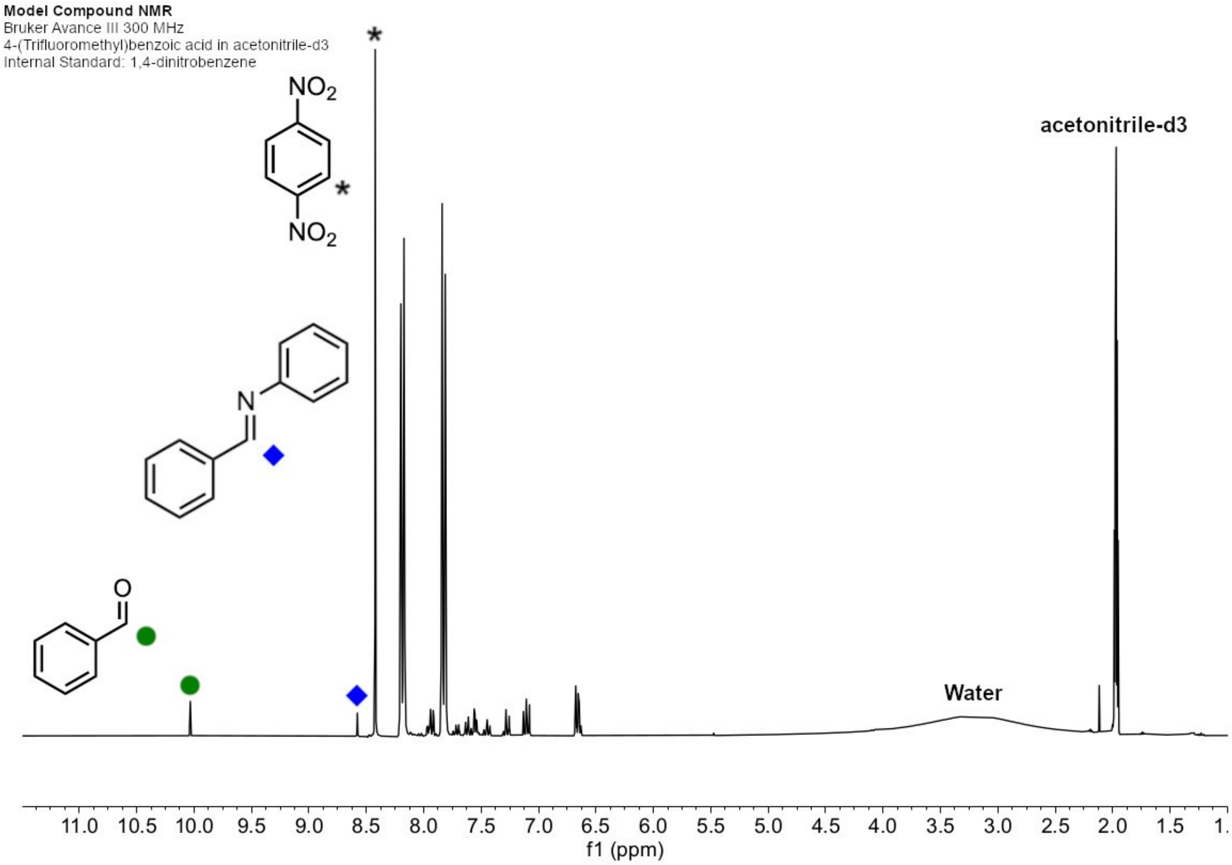

Figure S36. ^{1}H NMR spectrum of aniline + benzaldehyde + 4-(trifluoromethyl)benzoic acid (model compound reaction) in acetonitrile-d3.

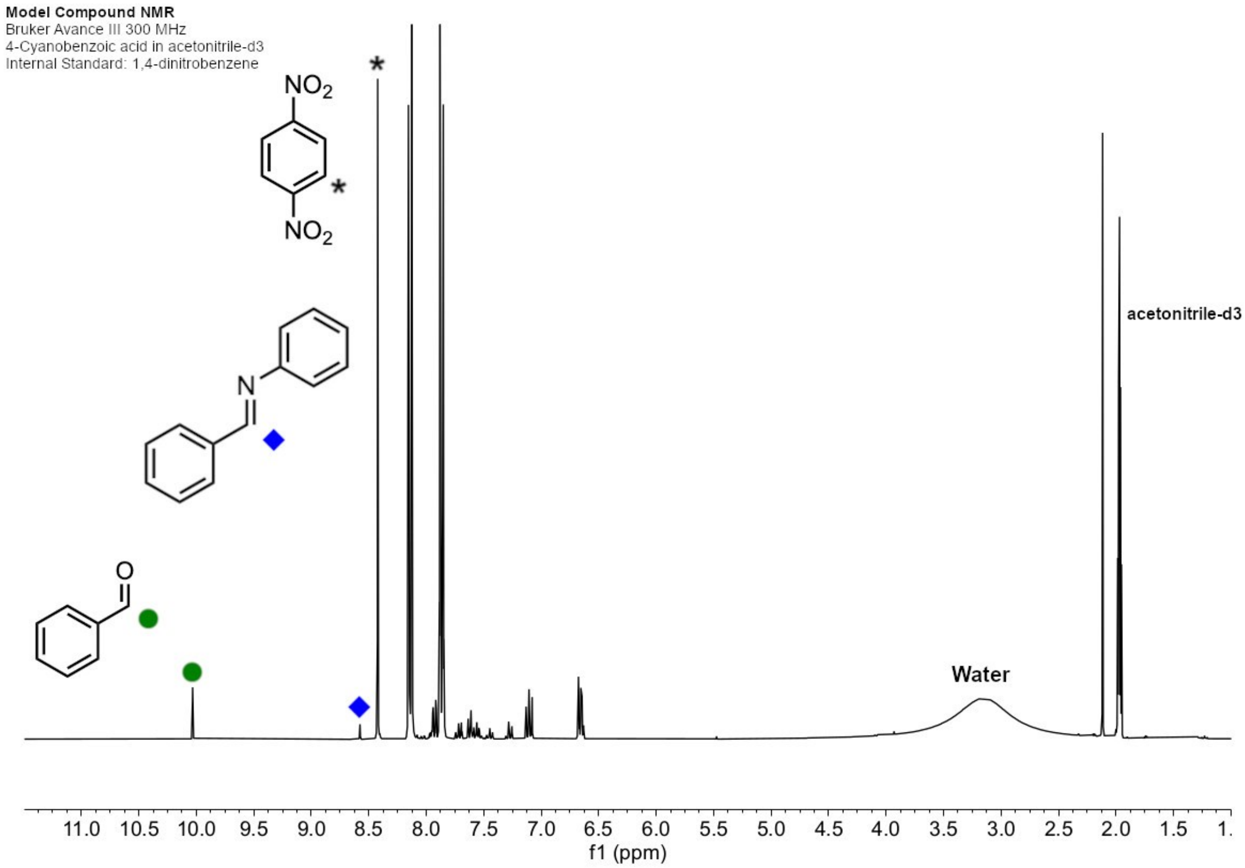

Figure S37. ^{1}H NMR spectrum of aniline + benzaldehyde + 4-cyanobenzoic acid (model compound reaction) in acetonitrile-d3.

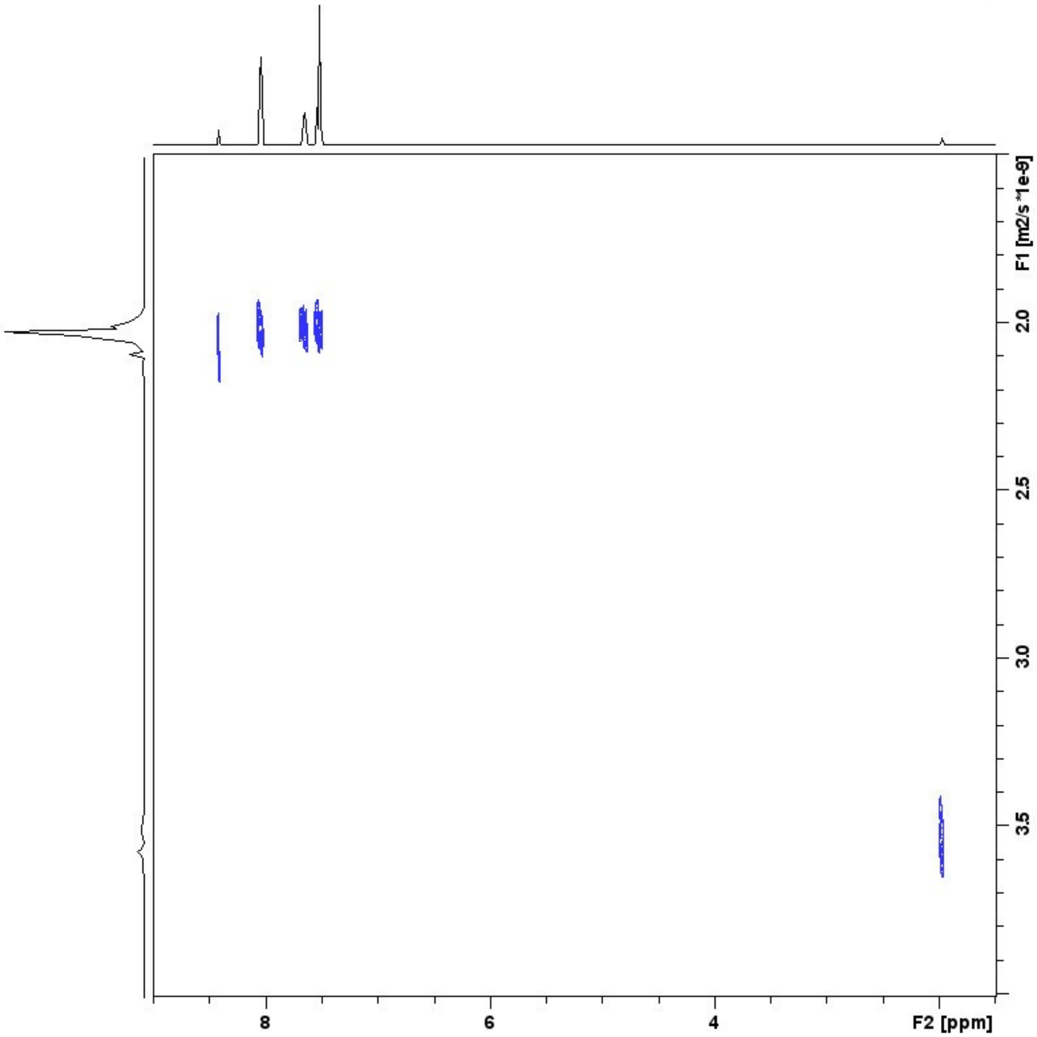

Figure S38. ^{1}H DOSY spectrum at 400 MHz of benzoic acid in acetonitrile-d3.

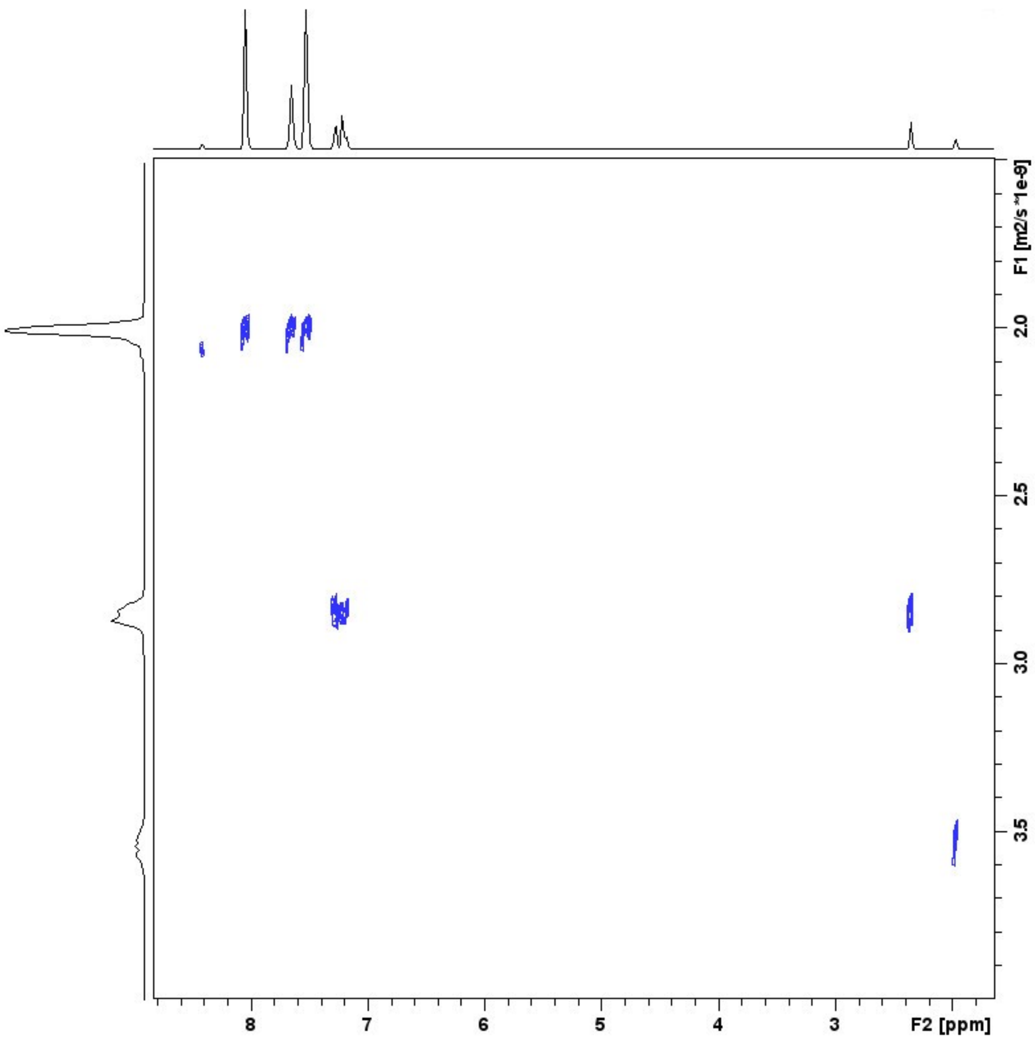

Figure S39. ^{1}H DOSY spectrum at 400 MHz of COF-300 + benzoic acid in acetonitrile-d3.

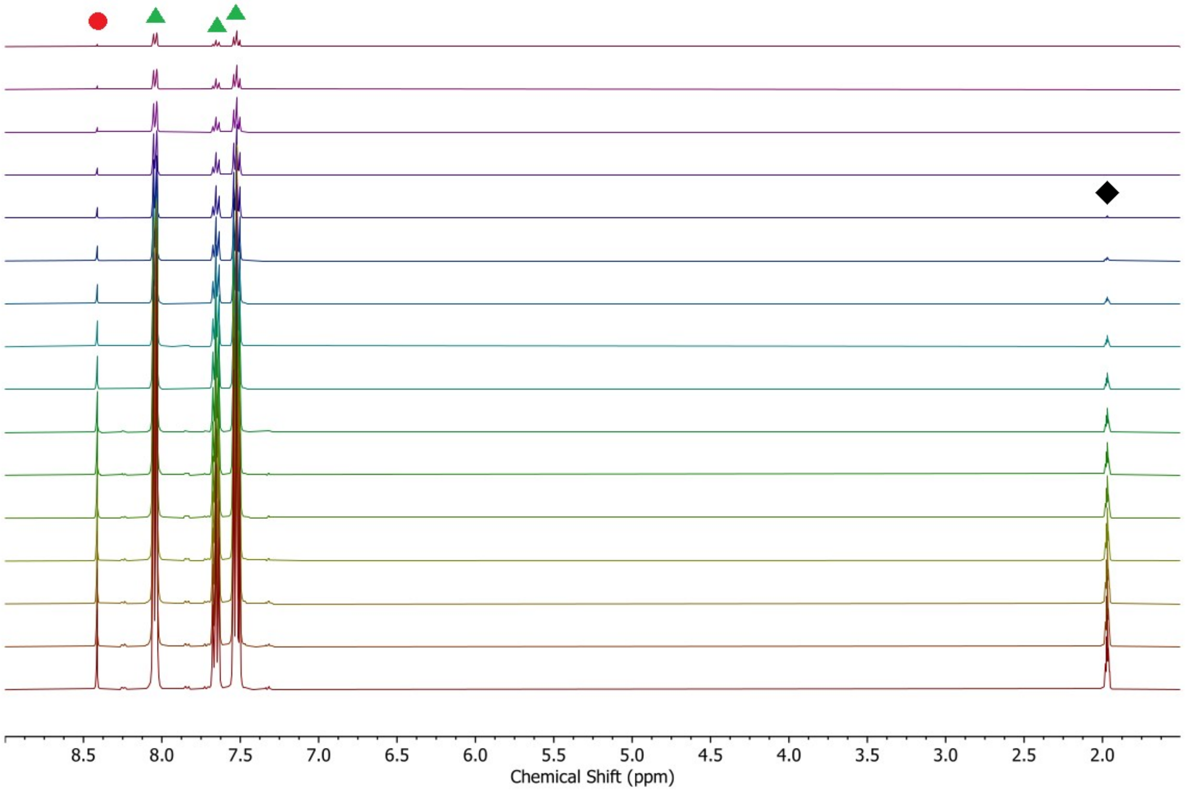

Figure S40. Stacked ^1H NMR spectra at 400 MHz of benzoic acid (green triangles) + 1,4-dinitrobenzene (red circles) in acetonitrile-d3 (black diamonds). Stacked spectra correspond to linearly varied gradient field strengths from 5 to 95% of the probe's maximum value in 16 steps. Acquisition parameters were optimized to be $\Delta = 0.0399$ s and $\delta = 800$ μs.

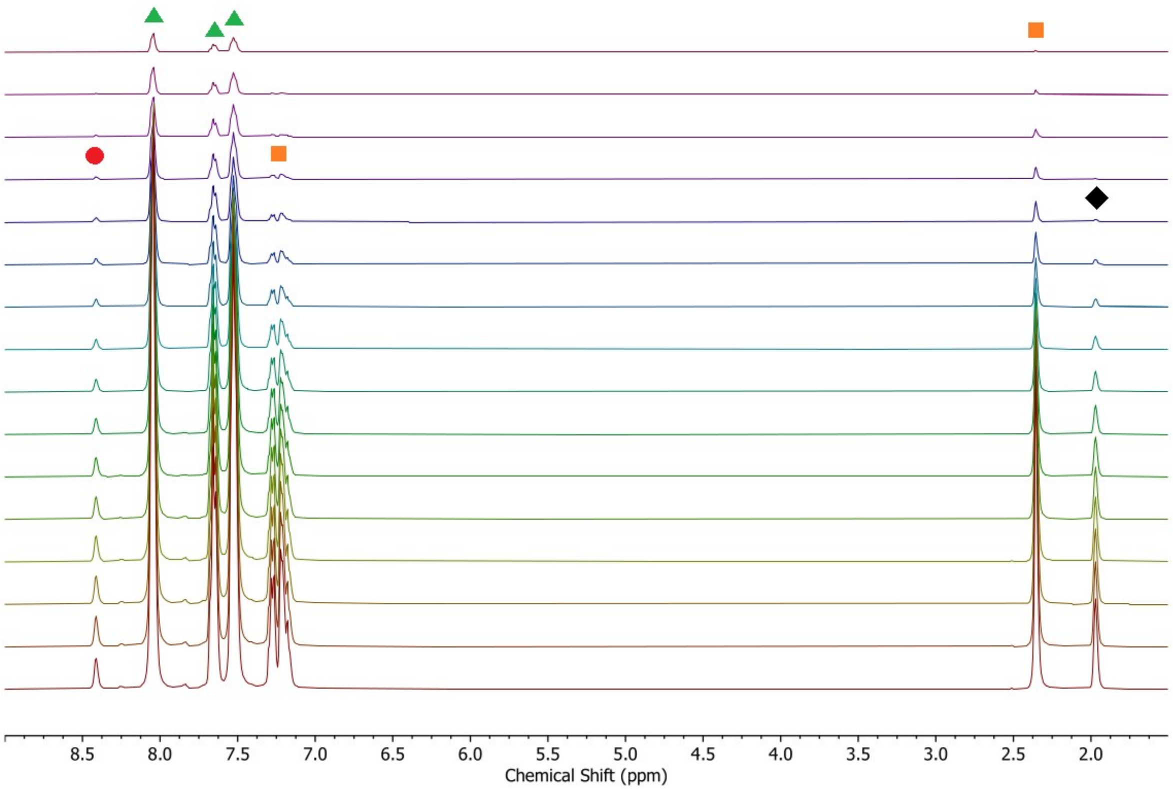

Figure S41. Stacked ^{1}H NMR spectra at 400 MHz of COF-300 + benzoic acid (green triangles) + 1,4-dinitrobenzene (red circles) + toluene (orange squares) in acetonitrile-d3 (black diamonds). Stacked spectra correspond to linearly varied gradient field strengths from 5 to 95% of the probe's maximum value in 16 steps. Acquisition parameters were optimized to be $\Delta = 0.0399$ s and $\delta = 800$ μs.

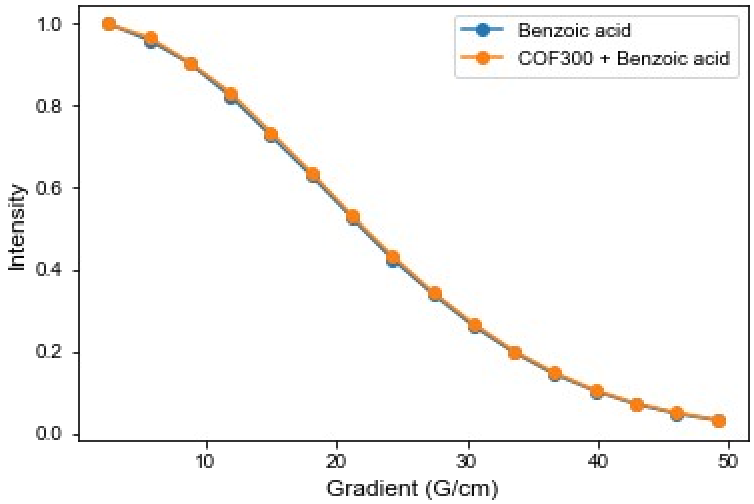

Figure S42. DOSY diffusion decay curves for benzoic acid in the presence and absence of COF-300 with respect to gradient field strength.

E. References

1. Ashiotis, G.; Deschildre, A.; Nawaz, Z.; Wright, J. P.; Karkoulis, D.; Picca, F. E.; Kieffer, J., *J Appl Crystallogr* **2015,** *48*, 510-519.
2. Juhás, P.; Davis, T.; Farrow, C. L.; Billinge, S. J. L., *Journal of Applied Crystallography* **2013,** *46*, 560-566.
3. Farrow, C. L.; Juhas, P.; Liu, J. W.; Bryndin, D.; Bozin, E. S.; Bloch, J.; Proffen, T.; Billinge, S. J., *J Phys Condens Matter* **2007,** *19*, 335219.
4. Uribe-Romo, F. J.; Hunt, J. R.; Furukawa, H.; Klock, C.; O'Keeffe, M.; Yaghi, O. M., *J. Am. Chem. Soc.* **2009,** *131*, 4570-4571.
5. Ma, T.; Kapustin, E. A.; Yin, S. X.; Liang, L.; Zhou, Z.; Niu, J.; Li, L.-H.; Wang, Y.; Su, J.; Li, J.; Wang, X.; Wang, W. D.; Wang, W.; Sun, J.; Yaghi, O. M., *Science* **2018,** *361*, 48-52.
6. Prill, D.; Juhás, P.; Schmidt, M. U.; Billinge, S. J. L., *Journal of Applied Crystallography* **2015,** *48*, 171-178.
7. Rademacher, N.; Daemen, L. L.; Chronister, E. L.; Proffen, T., *Journal of Applied Crystallography* **2012,** *45*, 482-488.
